# Acetyl-leucine slows disease progression in lysosomal storage disorders

**DOI:** 10.1093/braincomms/fcaa148

**Published:** 2020-12-20

**Authors:** Ecem Kaya, David A Smith, Claire Smith, Lauren Morris, Tatiana Bremova-Ertl, Mario Cortina-Borja, Paul Fineran, Karl J Morten, Joanna Poulton, Barry Boland, John Spencer, Michael Strupp, Frances M Platt

**Affiliations:** 1 Department of Pharmacology, University of Oxford, Oxford OX1 3QT, UK; 2 Department of Neurology, Inselspital, Bern University Hospital, University of Bern, 3010 Bern, Switzerland; 3 Department of Chemistry, School of Life Sciences, University of Sussex, Brighton, BN1 9RH UK; 4 Population, Policy and Practice Research and Teaching Department, Great Ormond Street Institute of Child Health, University College London, London WC1N 1EH, UK; 5 Nuffield Department of Women’s and Reproductive Health, University of Oxford, John Radcliffe Hospital OX3 9DU, Oxford, UK; 6 Department of Pharmacology and Therapeutics, Western Gateway Building, College of Medicine and Health, University College Cork, Cork, T12XF62, Ireland; 7 Department of Neurology and German Center for Vertigo and Balance Disorders, Ludwig Maximilians University, Munich, 81377 München, Germany

**Keywords:** lysosomal storage diseases, Niemann-Pick disease type C, GM2 gangliosidosis, acetyl-leucine, miglustat

## Abstract

Acetyl-dl-leucine is a derivative of the branched chain amino acid leucine. In observational clinical studies, acetyl-dl-leucine improved symptoms of ataxia, in particular in patients with the lysosomal storage disorder, Niemann-Pick disease type C1. Here, we investigated acetyl-dl-leucine and its enantiomers acetyl-l-leucine and acetyl-d-leucine in symptomatic *Npc1^−/−^* mice and observed improvement in ataxia with both individual enantiomers and acetyl-dl-leucine. When acetyl-dl-leucine and acetyl-l-leucine were administered pre-symptomatically to *Npc1^−/−^* mice, both treatments delayed disease progression and extended life span, whereas acetyl-d-leucine did not. These data are consistent with acetyl-l-leucine being the neuroprotective enantiomer. Altered glucose and antioxidant metabolism were implicated as one of the potential mechanisms of action of the l-enantiomer in *Npc1^−/−^* mice. When the standard of care drug miglustat and acetyl-dl-leucine were used in combination significant synergy resulted. In agreement with these pre-clinical data, when Niemann-Pick disease type C1 patients were evaluated after 12 months of acetyl-dl-leucine treatment, rates of disease progression were slowed, with stabilization or improvement in multiple neurological domains. A beneficial effect of acetyl-dl-leucine on gait was also observed in this study in a mouse model of GM2 gangliosidosis (Sandhoff disease) and in Tay-Sachs and Sandhoff disease patients in individual-cases of off-label-use. Taken together, we have identified an unanticipated neuroprotective effect of acetyl-l-leucine and underlying mechanisms of action in lysosomal storage diseases, supporting its further evaluation in clinical trials in lysosomal disorders.

## Teaser

This study investigates the disease-modifying effect of acetyl-DL-leucine in two neurodegenerative lysosomal storage diseases with different aetiologies: Niemann-Pick type C and GM2 gangliosidosis in both murine models and clinical studies. Acetyl-DL-leucine and its L enantiomer provide neuroprotection likely by altering metabolism – a new therapeutic approach for these diseases.

## Graphical Abstract

**Graphical Abstract:**
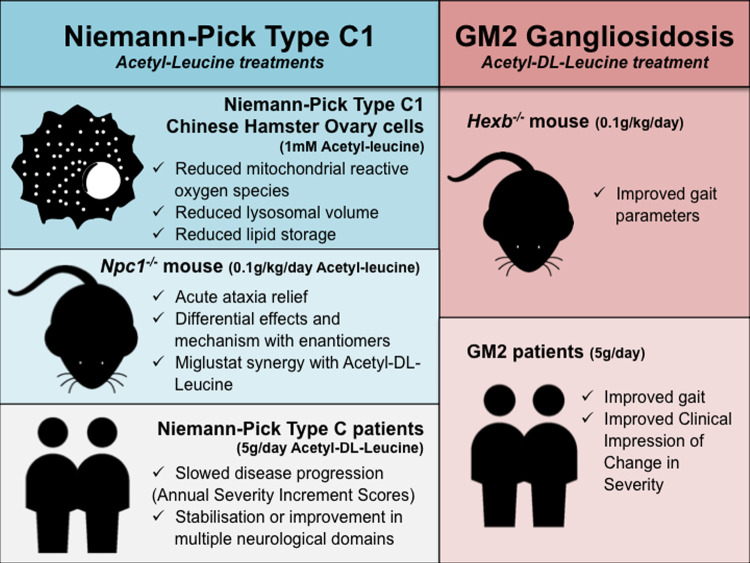

## Introduction

Acetyl-dl-leucine (ADLL) is a racemic (1:1) mixture of enantiomers and a derivative of the branched chain amino acid leucine. It has been used as a medication (Tanganil™) for the treatment of acute vertigo in France and former French colonies since 1957; it is orally available and has a good safety profile according to the Periodic Updated Safety Reports from Pierre-Fabre, France. Although its mechanism of action (MOA) is not fully known, studies in a guinea pig model of acute vertigo showed that ADLL can restore membrane potential and excitability of abnormally polarized neurons of the medial vestibular nucleus (Vibert and Vidal, 2001).

Due to the phylogenetic similarities and close interactions between vestibular and deep cerebellar neurons (Highstein and Holstein, 2005), and an activation of cerebellar neurons is an essential part of central vestibular compensation, the effects of ADLL were evaluated in patients with cerebellar ataxias who have impaired function of cerebellar neurons. ADLL was shown to be beneficial in patients with cerebellar ataxia (Strupp *et al.*, 2013; Schniepp *et al.*, 2016). Since cerebellar ataxia is one of the leading clinical symptoms of some lysosomal storage diseases (LSDs) such as Niemann-Pick disease type C1 (NPC1) and the GM2 gangliosidoses (Tay-Sachs and Sandhoff disease), the symptomatic effects of ADLL were examined in 12 patients with NPC1 who were treated with the oral formulation of ADLL for a month (5 g/day). ADLL therapy in NPC1 patients led to significant improvements in cerebellar ataxia as well as in cognition and behaviour, suggestive of potential functional benefits beyond the cerebellum (Bremova *et al.*, 2015). Our findings of *in vitro* benefit of ADLL in Tangier disease fibroblasts (Colaco *et al.*, 2019) and *in vivo* slowing disease progression in Sandhoff disease mice (Kaya *et al.*, 2020) suggest that ADLL may have the potential for therapeutic use in a broader range of diseases.

In all of these studies, ADLL was administered to patients as a racemic mixture of acetyl-d-leucine (ADL) and acetyl-l-leucine (ALL) enantiomers. Pre-clinical studies implicated ALL as the active isomer responsible for postural compensation after unilateral vestibular damage in an animal model (Günther *et al.*, 2015; Tighilet *et al.*, 2015), and three clinical trials are ongoing with ALL: NPC, GM2 gangliosidosis and Ataxia-Telangiectasia (clinicaltrials.gov NCT03759639, NCT03759665 and NCT03759678). CNS exposure of the administered compounds is equivalent within the limitations of the methods used, but l is metabolized rapidly whereas d is inert (Churchill *et al.*, 2020). However, it remains to be determined if this proposed MOA is relevant to the effects of ADLL and individual enantiomers in NPC.

In the current study, we have therefore investigated the efficacy of ADLL and its distinct enantiomer components in a mouse model of NPC1 (*Npc1^−/−^*). We found that ADLL and its enantiomers significantly improved ataxia in *Npc1^−/−^* mice, in agreement with clinical observations with ADLL (Bremova *et al.*, 2015). It is important to note that, when administered pre-symptomatically, ADLL and ALL slowed the rate of disease progression, were neuroprotective and significantly extended lifespan. In contrast, ADL did not have these effects. Therefore, pre-clinical studies implicate ALL as the active enantiomer of ADLL responsible for the neuroprotective effects of the treatment.

When miglustat, the currently approved therapy for NPC1 (Patterson *et al.*, 2007), was combined with ADLL in *Npc1^−/−^* mice, significant synergistic benefit resulted, including further extension of life span. In a cohort of NPC1 patients, the significant neuroprotective effects of ADLL identified in *Npc1^−/−^* mice were also observed in a 12–18-month extension phase of an ongoing observational study (Cortina-Borja *et al.*, 2018). When the MOA of ADLL and its enantiomers were investigated in the cerebellum of treated *Npc1^−/−^* mice, modulation of pathways involved in glucose metabolism was identified as potentially mediating the beneficial effects of the l-enantiomer. Finally, in individual-cases of off-label-use ADLL improved function in three GM2 gangliosidosis patients (Tay-Sachs and Sandhoff disease) and gait improvements were also demonstrated in a mouse model of Sandhoff disease. These findings demonstrate the distinct benefits of acetyl-leucine (AL) treatments in LSDs and show promise for clinical applications.

## Materials and methods

### Animals and treatments

BALBc/NPC1^nih^ (Pentchev *et al.*, 1980) and Sandhoff (*Hexb^−/−^*) mice (Sango *et al.*, 1995) were bred as heterozygotes to generate homozygous null (*Npc1^−/−^*) and mutant (*Hexb^−/−^*) mice, along with their respective wild-type controls (*Npc1^+/+^*, *Hexb^+/+^*). Mice were housed under non-sterile conditions, with food and water available *ad libitum*. Since the phenotypic development affects both genders similarly upon *Npc1* abrogation (Morris *et al.*, 1982), all experiments were conducted on female mice to facilitate group housing from different litters using protocols approved by the UK Home Office Animal Scientific Procedures Act, 1986. All animal works was conducted under the UK Home Office licencing authority and the Project licence number is P8088558D. Mice were randomly assigned to treatment groups and the drugs coded and the staff blinded to treatment when performing behavioural analysis.

### Chinese hamster ovary cells

Wild-type and NPC1-deficient Chinese hamster ovary (CHO) cells were used for *in vitro* experiments and have been described previously (Dahl *et al.*, 1992; Higaki *et al.*, 2001).

### Drug treatments

AL analogues and miglustat treatment protocols are described in the Supplementary experimental procedures.

### Mouse behavioural analysis

The weight and activity of each mouse were recorded weekly until reaching the humane endpoint (defined as a loss of 1 g body weight within 24 h). CatWalk [10.5 system (Noldus)], NG Rota Rod for mice (Ugo Basile) was performed as described in the Supplementary experimental procedures.

### Biochemical and immunohistochemical analyses

#### Sample preparation

Mice were saline perfused under terminal anaesthesia. Tissues for biochemical analysis were snap-frozen on ice-cold isopentane. For immunofluorescent staining, tissues were perfused with 4% paraformaldehyde followed by phosphate buffered saline (Gibco #14190144) and kept in 4% paraformaldehyde for 24 h then stored in phosphate buffered saline containing 20% w/v sucrose. Biochemical analyses were performed on water-homogenized tissues (50 mg/ml) and protein content determined (BCA protein assay, Thermo Fisher #23227) according to the manufacturer’s instructions.

#### Western blot analyses

Western blot analyses were performed on mouse cerebellum as described in the Supplementary experimental procedures.

#### Adenosine diphosphate and ATP extraction

Adenosine diphosphate (ADP) and ATP were extracted with Phenol-TE from 70 to 150 mg tissues according to published methods (Chida *et al.*, 2012). For details, see Supplementary experimental procedures.

#### Nicotinamide adenine dinucleotide and its reduced form extraction

Nicotinamide adenine dinucleotide (NAD) and its reduced form (NADH) extractions were performed on 20 mg phosphate buffered saline washed fresh tissue on wet ice homogenized with a Dounce homogenizer with 400 μl of NADH/NAD extraction buffer (Abcam NAD/NADH Assay kit #ab65348). For details, see Supplementary experimental procedures.

### Sample analysis

#### Sphingoid base measurements

Sphingoid base extraction and detection with reverse phase high performance liquid chromatography was performed as described in the Supplementary experimental procedures.

#### Glycosphingolipid measurements

Glycosphingolipids (GSLs) were extracted and measured by Normal Phase-high performance liquid chromatography according to published methods (Neville et al., 2004). For details, see Supplementary experimental procedures.

#### Cholesterol measurements

Cholesterol was measured from Folch-extracted tissues with the Amplex red Cholesterol assay kit according to the manufacturer’s instructions. For details, refer to Supplementary experimental procedures.

#### Flow cytometry experiments of CHO cells

Relative acidic compartment volume staining of live cells was with LysoTracker™ Green DND-26 (Thermo Fisher #L7526), mitochondrial volume was determined with MitoTracker Green (Invitrogen #M7514) and mitochondrial reactive oxygen species with MitoSOX Red (Invitrogen #M36008). For details, refer to Supplementary experimental procedures.

#### Filipin staining of CHO cells

Free unesterified cholesterol was visualized in CHO cells grown on glass coverslips using filipin labelling [from Streptomyces filipinensis (Sigma)]. For details, refer to Supplementary experimental procedures.

#### Immunohistochemistry

Immunohistochemistry was performed as described in the Supplementary experimental procedures. The antibodies used are summarized in Supplementary Table 1. Slides were air dried and mounted in ProLong Gold antifade (Invitrogen, #P36930).

#### Western blotting

Western blotting was performed as described in Supplementary experimental procedures. The antibodies used are summarized in Supplementary Table 1.

#### Image acquisition and quantification

Imaging of brain sections and CHO cells was performed with a Leica-SP8 confocal microscope. Western blot data acquisition was conducted with LiCOR Odyssey Infrared imaging system (Model No. 9120) and Universal hood II (Bio-Rad, California, USA). Mean fluorescence values, cerebellum diameters, areas and cell quantifications were calculated with Fiji Version 1.51g software (Image J) (http://fiji.sc/Fiji) (W. Rasband, NIH, USA). See Supplementary experimental procedures for analysis of cerebellar sections.

#### ADP/ATP measurements

Measurements of ADP/ATP were made using a kit from Sigma Aldrich (#MAK135) according to the manufacturer’s instructions. The ADP/ATP ratio was calculated with the equation: (RLU-C-RLUB)/RLU-A.

#### NAD, NADH measurements

NAD, NADH and total NAD (NADt) (NAD + NADH = NADt) were measured with the NAD/NADH assay kit (Abcam #ab65348). For details, refer to Supplementary experimental procedures.

### Clinical studies

*Demographics and statistical analysis of individual-cases of off-label-use of adult NPC1 patients treated with ADLL is* described in the Supplementary experimental procedures.

*Individual-cases of off-label-use in GM2 gangliosidosis patients* are described in the Supplementary experimental procedures.

*Blinded video-rating* is described in the Supplementary experimental procedures.

All patients gave their informed consent according to the Declaration of Helsinki prior to the compassionate use study.

### Statistical analysis and power calculations

To calculate the number of mice needed for the experimental groups in this study, we used G*Power software (http://www.gpower.hhu.de). The power was set as 0.8 and the significance level, *α*, as 0.05. The mean lifespan of the NPC1 mouse model in our facility is 87 days with a standard deviation of 3 days. NPC1 is a disease with no curative treatment and many experimental disease-modifying drugs have reported a 10–30% increase in lifespan. Therefore, we based our power calculation on a 10–30% effect size and a sample size of minimum five and maximum eight animals per group was determined. For statistical analysis, see Supplementary experimental procedures.

### Data availability

Data will be made available upon reasonable request.

## Results

### ADLL, ALL and ADL administered during the symptomatic phase of disease improves ataxia in *Npc1^−/−^* mice

**Figure 1:**
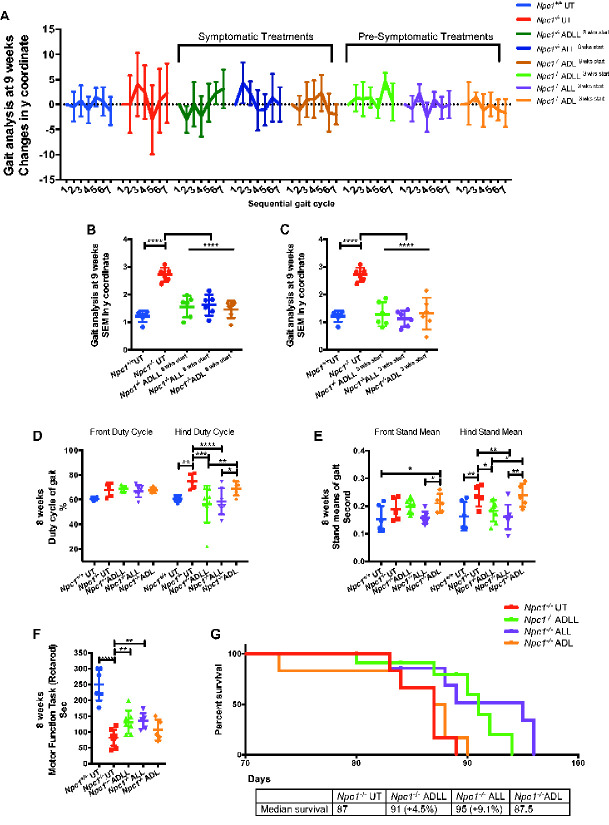
**AL analogues impact on behavioural parameters in NPC1 mice.** For wild-type untreated (*Npc1^+/+^* UT), NPC1 untreated (*Npc1^−/−^* UT), ADLL (*Npc1^−/−^* ADLL), ALL (*Npc1^−/−^* ALL), ADL (*Npc1^−/−^* ADL) treatments minimum five, maximum seven animals for each group. *P < 0.05, **P < 0.01, ***P < 0.001 and ****P < 0.0001. (**A**) Symptomatic and pre-symptomatic AL treatment *y* coordinates displacement of each consecutive foot from a straight-line trajectory (mean ± SD, *n* = 6). (**B**) Late stage AL treatment SEMs of *y* coordinate changes (mean ± SD, *n* = 6; one-way ANOVA. (**C**) Early stage AL treatment SEMs of *y* coordinate changes (mean ± SD, *n* = 6; one-way ANOVA). (**D**) Early stage AL treatment front and hind duty cycle measurements, mean ± SD (two-way ANOVA). (**E**) Early stage AL treatments front and hind stand mean measurements, mean ± SD (two-way ANOVA). Duty Cycle and Stand mean analyses were measured by three-recorded runs per animal and quantitative results were obtained with Noldus CatWalk 10.5 software system. (**F**) Early stage AL treatment motor performance measurements, mean ± SD (one-way ANOVA). Motor function performance was measured with accelerating Rotarod (1 rpm up to 10 rpm). (**G**) Life expectancy percentages and median survivals (Gehan–Breslow–Wilcoxon test), *n* = 6

*Npc1^−/−^* mice have a 10–12-week life span, with onset of symptoms (gait abnormalities, tremor and weight loss) beginning at 6–7 weeks of age (Williams *et al.*, 2014). ADLL, ALL and ADL were administered to *Npc1^−/−^* mice in their diet (0.1 g/kg/day), with a dose identical to that used in observational clinical studies (Bremova *et al.*, 2015). Untreated 9-week-old *Npc1^−/−^* mice exhibit statistically significant ataxia that presents as a pronounced sigmoidal gait, relative to wild-type mice (*P* < 0.0001) (Supplementary Fig. 1), whereas 9-week-old *Npc1^−/−^* mice treated with ADLL, ALL or ADL from 8 weeks of age (1 week of treatment) displayed significantly reduced ataxia as determined by measuring lateral displacement from a straight trajectory in an automated gait analysis system (Supplementary Fig. 1) (Fig. 1A and B) (*P* < 0.0001, all treatments). These results indicate that the acute anti-ataxic effect of AL is stereoisomer independent in *Npc1^−/−^* mice.

### Pre-symptomatic treatment with ADLL, ALL and ADL improves ataxia in *Npc1*^*−/−*^ mice

We investigated whether any additional benefit was conferred by AL analogues if treatment commenced before symptom onset. *Npc1^−/−^* mice were therefore treated pre-symptomatically from weaning (3 weeks of age) and assessed at 9 weeks of age (6 weeks of treatment). Similar to the acute treatment response, all three AL analogues tested significantly reduced ataxia (Fig. 1A and C) (*P* < 0.0001 for all treatments) with the magnitude comparable to the effects observed with 1 week of treatment (Fig. 1A and Supplementary Fig. 1).

### Pre-symptomatic treatment with ADLL and ALL, but not ADL, improves gait abnormalities, motor function and modestly extends survival in *Npc1*^*−/−*^ mice

*Npc1^−/−^* mice develop motor dysfunction (measured by Rotarod) and paw placement abnormalities when ambulatory (measured with CatWalk). We therefore treated *Npc1^−/−^* mice pre-symptomatically from weaning (3 weeks of age) and assessed the mice at 8 weeks of age. Untreated *Npc1^−/−^* mice had a significantly improved hind paw duty cycle (Fig. 1D, *P* = 0.0031) and an improved hind paw stand mean (*P* = 0.0026) relative to wild-type mice (Kirkegaard *et al.*, 2016) (Fig. 1E). Pre-symptomatic ADLL and ALL treatment of *Npc1^−/−^* mice resulted in a functional improvement in duty cycle of hind paws (ADLL: *P* = 0.0005, ALL: *P* < 0.0001) (Fig. 1D) and hind paw stand mean (ADLL: *P* = 0.0179, ALL: *P* = 0.0014) relative to untreated *Npc1^−/−^* mice (Fig. 1E). Rotarod performance was significantly impaired in untreated *Npc1^−/−^* mice relative to wild-type littermates (*P* < 0.0001) (Fig. 1F). Treatment with ADLL (*P* = 0.0088) and ALL (*P* = 0.0070) resulted in a significant improvement in Rotarod performance by *Npc1^−/−^* mice (Fig. 1F). ADL treatment had no significant benefit on *Npc1^−/−^* mouse motor function, as assessed using CatWalk (hind paw duty cycle *P* = 0.2124, hind paw stand mean *P* = 0.9547) or Rotarod (*P* = 0.2218) (Fig. 1D–F). Relative to untreated *Npc1^−/−^* mice, the life span of animals treated from weaning was modestly but significantly increased by 8 days (9.1%) with ALL treatment (*P* = 0.0334), 4 days with ADLL (*P* = 0.0305) (4.5%) and was not changed with ADL (*P* = 0.6908) (Fig. 1G), therefore displaying l-enantiomer selectivity.

### ADLL, ALL and ADL improve neuropathology and reduce some lipid species in *Npc1*^*−/−*^ mouse brain in a stereo-selective manner

**Figure 2:**
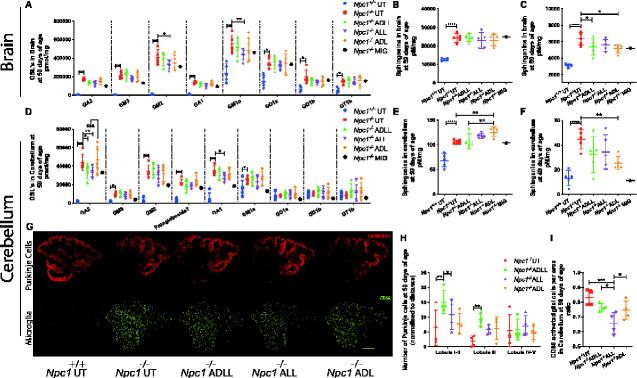
**Effect of AL analogues on biochemical and histopathology in *Npc1^−/−^* mice at 59 days of age.** For all AL treatments, *n* = 5 animals per group, for miglustat (positive control) (*Npc1^−/−^* MIG), *n* = 2 or *n* = 1. *P < 0.05, **P < 0.01, ***P < 0.001 and ****P < 0.0001. (**A**) GSL profiles in brain, mean ± SD (two-way ANOVA). (**B**) Sphingosine levels in brain, mean ± SD (one-way ANOVA). (**C**) Sphinganine levels in brain, mean ± SD (one-way ANOVA). (**D**) GSLs in cerebellum, mean ± SD (two-way ANOVA). (**E**) Sphingosine in cerebellum, mean ± SD (one-way ANOVA). (**F**) Sphinganine in cerebellum, mean ± SD (one-way ANOVA). (**G**) Cerebellum stained with calbindin-b (Purkinje cells, red) or CD68 (activated microglia, yellow). Scale bar 800 μm. (**H**) Purkinje cell density (Purkinje cell number normalized by perimeter of each lobule) at 59 days of age relative to NPC1 untreated, mean ± SD (two-way ANOVA). (**I**) CD68 cell density (number of CD68 positive microglia per area) at 59 days of age, mean ± SD (one-way ANOVA).

Since NPC1 disease is characterized by the accumulation of sphingoid bases (sphingosine and sphinganine), cholesterol, sphingomyelin, free fatty acids and GSLs (Lloyd-Evans and Platt, 2010), we measured the impact of ALs administered to *Npc1^−/−^* mice from 3 weeks of age on lipid storage in the brain. At 59 days of age, *Npc1^−/−^* mice exhibited increased lipid levels relative to wild-type (Williams *et al.*, 2014) (Fig. 2A–F). In order to evaluate the differential impact of the AL analogues in the CNS, we analysed the cerebellum and the forebrain (referred to as brain in Fig. 2) separately. Total GSLs in the forebrain were not significantly altered by any of the AL treatments (Supplementary Fig. 2A), while ALL selectively reduced GM1a (20.1%; *P =* 0.0018) and GM2 (19.6%; *P =* 0.0222) (Fig. 2A). Interestingly, although sphingosine levels were not significantly affected, ADLL and ADL reduced sphinganine levels by 13.5% (*P =* 0.0456) and 18.2% (*P =* 0.0111), respectively (Fig. 2B and C). Analysis of the cerebellum confirmed no significant difference in total GSLs relative to untreated *Npc1^−/−^* mice with any of the AL analogues tested (Supplementary Fig. 2B). However, ADLL and ALL treatment significantly lowered levels of specific GSLs including GA2 (ADLL: 21.3%, *P =* 0.0102; ALL: 23.8%, *P* = 0.0042), whereas a significant reduction in GA1 was only observed with ALL treatment (23.5%, *P =* 0.0254) (Fig. 2D). Sphingosine levels were increased by 19%, (*P =* 0.0042), but sphinganine was significantly reduced following ADL treatment (42.3%, *P =* 0.0074) (Fig. 2E and F). These results indicate differential biological targets of the two enantiomers. Cholesterol levels in the forebrain and in the cerebellum were not shown as total brain cholesterol levels are not changed in the NPC brain (Xie *et al.*, 2000).

### ADLL slows Purkinje cell loss and ALL reduces neuroinflammation in the cerebellum, whereas ADL has no neuroprotective effect

*Npc1^−/−^* mice exhibit progressive neurodegeneration, with cerebellar Purkinje neuron loss progressing from anterior to posterior lobes, accompanied by microglial activation (Williams *et al.*, 2014) (Fig. 2G). Only ADLL treatment significantly increased Purkinje cell survival at 59 days of age relative to untreated *Npc1^−/−^* littermates: 133% more Purkinje cells were present in lobules I and II (*P =* 0.0027), and 402% more Purkinje cells in lobule III (*P =* 0.0108) (Fig. 2G and H). Other treatments did not significantly improve Purkinje cell survival in any cerebellar lobules (*Npc1^−/−^* ALL lobules I–II *P =* 0.107, lobule III *P =* 0.157, lobules IV–V *P =* 0.533; *Npc1^−/−^* ADL lobules I–II *P =* 0.60, lobule III *P =* 0.11, lobules IV–V *P =* 0.766, compared to *Npc1^−/−^* UT) (Fig. 2G and H). However, it is important to note that all of the *Npc1^−/−^* experimental groups (untreated–treated) possessed 5–10 times lower numbers of Purkinje cells in their cerebellum compared to wild-type mice (Supplementary Fig. 2C). In addition, only ALL treatment significantly reduced (by 20%, *P =* 0.0177) the frequency of CD68-positive activated microglia (Fig. 2G and I) while other treatments did not have a significant impact (*P =* 0.1353 *for Npc1^−/−^* ADLL, *P* = 0.0553 for *Npc1^−/−^* ADL compared to *Npc1^−/−^* UT). ADL did not mediate any long term, neuroprotective effects assessed with Purkinje cell count and CD68 staining. Lastly, we measured myelin basic protein levels in the cerebellum by western blotting and saw no changes in myelin basic protein content with AL treatments (Supplementary Fig. 2D and E).

### All AL treatments alleviate lipid storage in non-neuronal tissues and cells

**Figure 3:**
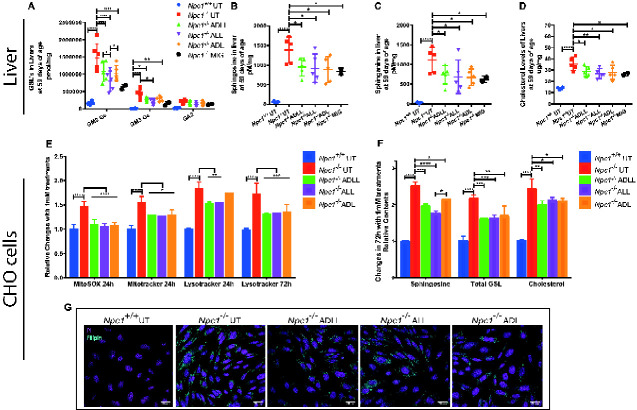
**Effect of AL analogues on non-neuronal tissues and cells.** For all AL treatments, *n* = 5 animals per group, for miglustat (positive control) (*Npc1^−/−^* MIG), *n* = 2 or *n* = 1. *P < 0.05, **P < 0.01, ***P < 0.001 and ****P < 0.0001. (**A**) GSLs in liver, mean ± SD (two-way ANOVA). (**B**) Sphingosine in liver, mean ± SD (one-way ANOVA). (**C**) Sphinganine in liver, mean ± SD (one-way ANOVA). (**D**) Liver cholesterol, mean ± SD (one-way ANOVA). (**E**) MitoSOX(red) and MitoTracker(Green) and Lysotracker(Green) changes relative to *Npc1*^+/+^ with 24 and 72 h 1 mM ALs. CHO cells were double stained with either MitoTracker Green and MitoSOX red, or LysoTracker Green and propidium iodide. Single and live cell populations and FITC and PE fluorescence were determined by analytical flow cytometry. Mean ± SD (two-way ANOVA). (**F**) Sphingosine, total GSL and free cholesterol levels (72 h) relative to *Npc1*^+/+^, mean ± SD (two-way ANOVA). (**G**) NPC1 CHO cells stained with filipin (cyan, cholesterol) and propidium iodide (magenta, nucleus). Scale bar 20 μm.

Liver GSL levels in the *Npc1^−/−^* mice were significantly reduced in *Npc1^−/−^* mice treated with ADLL (26.8%, *P =* 0.03), ADL (26.9%, *P =* 0.0253) and 45.5% for ALL (*P =* 0.0003) (Supplementary Fig. 2F). Quantification of the major GSL species confirmed that levels of GM2Gc (the most abundant GSL in the mouse liver) were decreased significantly with all AL analogues tested (26.7% ADLL: *P =* 0.0002, 45% ALL: *P <* 0.0001, 29% ADL: *P <* 0.0001), while GM3Gc levels were only reduced significantly by ALL (54.8%: *P =* 0.0314) relative to untreated *Npc1^−/−^* mice (Fig. 3A). All analogues tested caused significant and comparable levels of reduction of sphingosine (the catabolic break down product of ceramide) (ADLL 29.5%, *P =* 0.0233; ALL 33.2%, *P* = 0.0148; ADL 33.6% *P* = 0.0103) and its *de novo* precursor sphinganine (ADLL 32.5% *P =* 0.0365; ALL 37.4% *P* = 0.0189; ADL 38.5% *P* = 0.0163) (Fig. 3B and C). Total free cholesterol in *Npc1^−/−^* liver was also significantly decreased after treatment with ADLL, ALL or ADL (ADLL 18.2% *P =* 0.0346; ALL 23.4% *P* = 0.0091; ADL 16.2% *P* = 0.0210) (Fig. 3D).

Reduction of lipid species in the liver with all three AL treatments led us to investigate whether other non-neuronal cell types were corrected by ALs. We conducted a series of experiments on NPC1-deficient CHO cells by utilizing lysosomal and mitochondrial probes as well as measurements of lipid storage. NPC1 protein deletion/mutation results in increased lysosomal volume (Te Vruchte *et al.*, 2014), as well as an increase in mitochondria (measured by MitoTracker), mitochondrial superoxide increase (measured by MitoSOX) (Paulina Ordonez *et al.*, 2012). CHO cells treated with 1 mM ALs for 24 h resulted in normalization of MitoSOX staining with all three drugs (*P <* 0.0001, compared to NPC1 UT) (Fig. 3E and Supplementary Fig. 3), and a significant decrease in MitoTracker Green (ADLL 16.8% *P* = 0.0150; ALL 17.1% *P* = 0.0133; ADL 15.8% *P* = 0.0215) (Fig. 3E and Supplementary Fig. 3). We found that only 1 mM ADLL and ALL treatment reduced relative lysosomal volume after 24 h (ADLL 16.9%, *P =* 0.0137; ALL 16%, *P* = 0.0177) (Fig. 3E and Supplementary Fig. 3); however, this beneficial effect was seen with all three AL treatments after 72 h of treatment (ADLL 24% *P =* 0.0026; ALL 22.8% *P* = 0.0036; ADL 22.3% *P* = 0.0042) (Fig. 3E and Supplementary Fig. 3). After 72-h AL treatment, sphingosine levels were significantly reduced by 22% with ADLL (*P* = 0.0009), 29% with ALL (*P* < 0.0001) and 14% with ADL (*P* = 0.0149) (Fig. 3F). Total GSL levels were significantly reduced with all treatments (26% with ADLL *P* = 0.0007; 25% with ALL *P* = 0.0010; 22% with ADL *P* = 0.0024) (Fig. 3F). Likewise, cholesterol content was significantly reduced (18% with ADLL *P* = 0.0043; 13% with ALL *P* = 0.0263; 14% with ADL *P* = 0.0212) (Fig. 3F and G).

### ADLL synergizes with miglustat in *Npc1*^*−/−*^ mice

**Figure 4:**
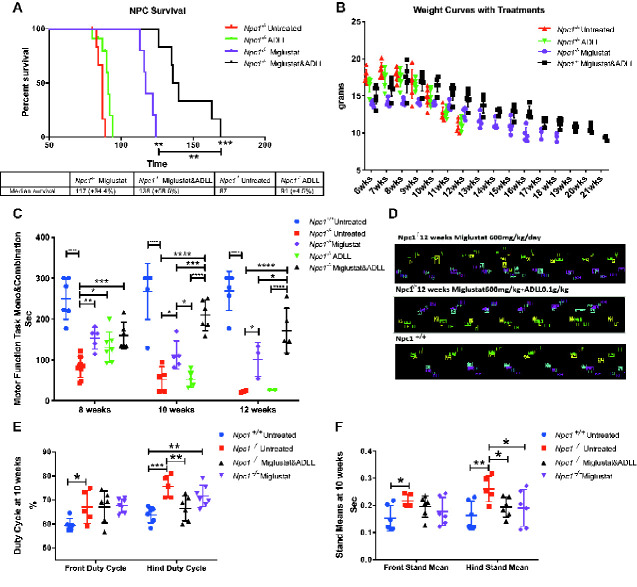
**Effect of ADLL in combination with miglustat.** For wild-type untreated (*Npc1^+/+^* UT), NPC1 untreated (*Npc1^−/−^* UT), ADLL (*Npc1^−/−^* ADLL), miglustat (*Npc1^−/−^* MIG) and combination (*Npc1^−/−^* miglustat and ADLL) therapies minimum five animals for each group. *P < 0.05, **P < 0.01, ***P < 0.001 and ****P < 0.0001. (**A**) Survival curves (percentage of life expectancy) and median survivals (Gehan–Breslow–Wilcoxon test). (**B**) Body weight curves of experimental groups over the course of their life, mean ± SD. (**C**) Motor performance measurements (Rotarod) at 8, 10 and 12 weeks of age, mean ± SD (two-way ANOVA). Animals that did not perform the task were excluded from the experiment. (**D**) Representative image of footprints of miglustat and miglustat/ADLL combination therapy relative to wild-type. (**E**) Duty cycle measurements of front and hind paws at 10 weeks of age, mean ± SD (two-way ANOVA). (**F**) Stand mean measurements at of front and hind paws 10 weeks of age, mean ± SD (two-way ANOVA)

We treated *Npc1^−/−^* mice with ADLL in combination with the standard of care drug miglustat (600 mg/kg/day), resulting in a statistically significant longer life span than animals on monotherapy (*Npc1^−/−^* miglustat versus *Npc1^−/−^* miglustat and ADLL *P =* 0.0016; *Npc1^−/−^* ADLL versus *Npc1^−/−^* miglustat and ADLL *P =* 0.0063; median survivals; *Npc1^−/−^* UT: 87 days, *Npc1^−/−^* ADLL: 91 days; *Npc1^−/−^* miglustat: 117 days, *Npc1^−/−^* miglustat and ADLL: 138 days) (Fig. 4A). Miglustat is known to cause weight loss through appetite suppression (Priestman *et al.*, 2008). When ADLL was used in combination with miglustat it prevented the weight loss associated with miglustat treatment (Fig. 4B) but not the weight loss resulting from progression of the disease. Combination therapy significantly increased Rotarod performance (8 weeks: *P =* 0.0007; 10, 12 weeks *P <* 0.0001 versus untreated *Npc1^−/−^*), and was associated with increased benefit relative to mice receiving either miglustat (10 weeks: *P =* 0.0299; 12 weeks: *P* = 0.0232) or ADLL monotherapy (10, 12 weeks: *P <* 0.0001) (Fig. 4C). Gait analysis at 10 weeks of age demonstrated that hind Duty Cycle percentages were significantly improved with combination therapy (*P =* 0.0028) whereas miglustat monotherapy showed no benefit (Fig. 4D and E). Stand mean significantly improved in mice treated with either combination therapy (*P =* 0.0250) or miglustat monotherapy (*P =* 0.0196) (Fig. 4D and F). Due to limited availability of miglustat, ALL and ADL combination therapies were not evaluated.

### Potential targets of leucine analogues in the cerebellum

**Figure 5:**
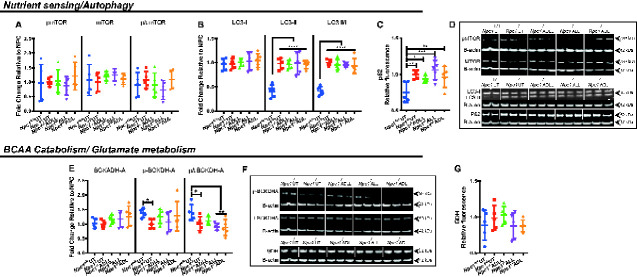
**Effects of AL analogues on expression of proteins involved in nutrient sensing, autophagy, branched chain amino acid catabolism and glutamate metabolism in cerebellum at 59 days of age.** Wild-type untreated (*Npc1^+/+^* UT), NPC1 untreated (*Npc1^−/−^* UT), ADLL (*Npc1^−/−^* ADLL), ALL (*Npc1^−/−^* ALL), ADL (*Npc1^−/−^* ADL) treatments, *n* = 5 for each group. *P < 0.05, **P < 0.01, ***P < 0.001 and ****P < 0.0001. (**A**) mTOR, Phospho-mTOR expression and phospho-mTOR and mTOR ratio, mean ± SD (two-way ANOVA). (**B**) LC3-I, LC3-II expression, LC3-II and LC3-I ratios, mean ± SD (two-way ANOVA). (**C**) p62 levels, mean ± SD (one-way ANOVA). (**D**) Western blots images of phospho-mTOR, mTOR, LC3 and p62 and their beta-actin loading controls. (**E**) BCKDHA, pPBCKDHA, p/t BCKDHA expression, mean ± SD, (2-way ANOVA). (**F**) BCKDHA, pBCKDHA, and glutamate dehydrogenase western blot images with beta-actin loading controls. (**G**) Glutamate dehydrogenase expression, mean ± SD (one-way ANOVA)

We investigated the potential targets that ALs could affect in the cerebellum since this brain region is particularly important in NPC1-related pathology. As l-leucine is a potent activator of the mammalian target of rapamycin (mTOR) (Kimball *et al.*, 1999), which negatively regulates autophagy (Klionsky and Emr, 2000), we investigated mTOR and its phosphorylation status in the cerebellum of AL-treated *Npc1^−/−^* mice. Western blotting indicated that levels of mTOR and its phosphorylation on serine 2448 (Yanagisawa *et al.*, 2017) [phosphorylated/total protein expression ratios (p/t)] were not changed by ADLL, ALL or ADL (Fig. 5A and D). Autophagic vacuoles accumulate in NPC1 disease, consistent with impaired lysosomal flux (Boland *et al.*, 2010). None of the compounds significantly changed the ratio of LC3-I and LC3-II (Fig. 5B and D). Furthermore, none of the ALs changed autophagic function or flux (Fig. 5B and D). In addition, we were unable to detect a significant change in levels of its substrate p62/SQSTM1(Pankiv *et al.*, 2007) (Fig. 5C and D).

### Effects on energy metabolism and antioxidant pathways

In order to investigate potential disruption of branched chain amino acid metabolism, we examined mitochondrial branched chain keto acid dehydrogenase enzyme A subunit (BCKDHA), which functions in the final step of the pathway that yields acetyl-CoA (Harris *et al.*, 2005). Phosphorylation of BCKDHA (that inactivates the enzyme) was significantly reduced in untreated *Npc1^−/−^* cerebellum relative to wild-type (37.6%, *P =* 0.0254) and the ratio of phosphorylated enzyme (p) and total enzyme (t), p/t, was significantly increased (36.6%, *P =* 0.0269) (Fig. 5E and F) in *Npc1^−/−^* cerebellum. None of the ALs tested had a significant impact on either total BCKDHA levels or the extent of its phosphorylation (Fig. 5E and F). Branched chain amino acids, especially leucine, are important for glutamine/glutamate balance and for energy metabolism when glucose metabolism is deficient or impaired (Yudkoff, 1997; Kennedy *et al.*, 2016). However, there was no difference in mitochondrial enzyme glutamate dehydrogenase protein levels between untreated *Npc1^−/−^* and *Npc1^+/+^* mice or following AL treatments (Fig. 5F and G).

**Figure 6:**
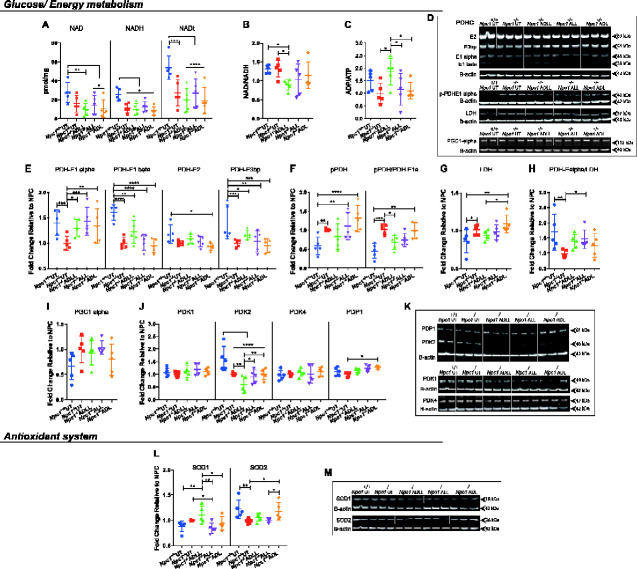
**Effects of AL analogues on expression of proteins involved in energy metabolism and the antioxidant system in cerebellum at 59 days of age.** Wild-type untreated (*Npc1^+/+^* UT), NPC1 untreated (*Npc1^−/−^* UT), ADLL (*Npc1^−/−^* ADLL), ALL (*Npc1^−/−^* ALL), ADL (*Npc1^−/−^* ADL) treatments, *n* = 5 for each group. *P < 0.05, **P < 0.01, ***P < 0.001 and ****P < 0.0001. (**A**) NAD, NADH, total NAD (NADt) measurements, mean ± SD (two-way ANOVA). (**B**) NADH/NADH ratios, mean ± SD (one-way ANOVA). (**C**) ADP/ATP ratios mean ± SD (one-way ANOVA). (**D**) Western blot images of PDHC, pPDH, LDH, peroxisome proliferator-activated receptor-gamma coactivator 1-alpha and beta-actin loading controls. (**E**) PDHC protein expressions, mean ± SD (two-way ANOVA). (**F**) pPDH expressions and pPDH/PDHE1 alpha ratios, mean ± SD (two-way ANOVA). (**G**) LDH expression, mean ± SD (one-way ANOVA). (**H**) PDHE1 alpha/LDH ratios, mean ± SD (one-way ANOVA). (**I**) PGC 1 alpha expression, mean ± SD (one-way ANOVA). (**J**) PDK 1–2–4 and PDP1 expression, mean ± SD (two-way ANOVA). (**K**) Western blot images of PDKs and PDP1 along with their beta-actin loading controls. (**L**) SOD1 and SOD2 expression, mean ± SD (two-way ANOVA). (**M**) Western blot images of SOD1 and SOD2 along with beta-actin loading controls

The ratio of NAD/NADH regulates various metabolic pathway enzymes, such as those involved in glycolysis and the tricarboxylic acid (TCA) cycle [including pyruvate dehydrogenase (PDH) enzyme] (Srivastava, 2016). NAD/NADH production (mostly derived from the TCA cycle) fuels the mitochondrial respiratory chain and therefore is of high importance in energy metabolism (Da Veiga Moreira *et al.*, 2015). We therefore measured the changes in NAD/NADH content in the cerebellum and their ratio upon AL treatments. NAD and NADH were decreased in *Npc1^−/−^* relative to *Npc1^+/+^* mice achieving statistical significance for NADH (55.1%, *P =* 0.0314) and the sum of NAD and NADH (49.2%, *P =* 0.0001) (Fig. 6A). Although there was no change in the levels of the coenzymes following AL treatments, in comparison with untreated NPC1 and WT (Fig. 6A), the NAD/NADH ratio was significantly decreased with ADLL treatment (30.7%, *P =* 0.0240) (Fig. 6B), which might be an indication of active TCA cycle machinery and glycolysis. The status of energy metabolism also can be assessed by determining ADP and adenosine triphosphate ratio (ADP/ATP). ADP/ATP ratio was also increased with ADLL (Fig. 6C) (*P =* 0.0101), which might indicate increased energy expenditure and a glycolytic state.

### ADLL increases active PDH levels while ALL shifts glucose metabolism towards PDH from lactate dehydrogenase

We then investigated the effect of AL analogues on glucose utilization that yields NAD (via lactate production) or NADH (via TCA cycle), by measuring levels of PDH and lactate dehydrogenase (LDH) proteins by western blotting (Fig. 6D–H). PDH is composed of three subunits: E1–3, with the active site located on the alpha subunit of E1. Phosphorylation of the E1 alpha subunit inactivates the PDH enzyme (Kato *et al.*, 2008). Untreated *Npc1^−/−^* mice at 59 days of age showed significantly decreased levels of PDH-E1 alpha (40.2%, *P =* 0.0009), PDH-E1 beta (61.2%, *P <* 0.0001), PDH-E3 binding protein (PDH-E3bp) (42.2%, *P =* 0.0005) (Fig. 6D and E), while phospho-PDH (inactive form) (41.4%, *P =* 0.0092), pPDH/PDH E1alpha ratio (55.5%, *P =* 0.0006) and LDH increased in the disease group (14.2%, *P =* 0.0335) (Fig. 6D, F and G), in comparison to WT. Accordingly, the glucose metabolism preference (PDHE1 alpha/LDH) favoured LDH dependency in untreated *Npc1^−/−^* mice (74.2%, *P =* 0.0060) with no detectable difference in proliferator-activated receptor-gamma coactivator 1-alpha levels (Fig. 6D–I). E1 alpha subunit protein levels in PDHC were significantly increased by ADLL (29.1%, *P* = 0.0146), ALL (43.3%, *P* = 0.0004) and ADL (34.17%, *P* = 0.0044) in the cerebellum from treated *Npc1^−/−^* mice (Fig. 6D and E), with no changes in El beta, E2 or E3bp. The protein levels of phosphorylated E1 alpha subunit were not significantly different following treatment compared to untreated *Npc1^−/−^* mice. However, the ratio of phosphorylated E1 alpha to total E1 alpha subunit was reduced with ADLL (33%, *P* = 0.0333) (Fig. 6D and F). LDH levels were the same in untreated and treated *Npc1^−/−^* mice (Fig. 6D and G). Nevertheless, the ratio of E1 alpha subunit to LDH was significantly shifted in favour of PDH by ALL (49.1%, *P* = 0.0494) (Fig. 6H). Finally, we measured the protein levels of peroxisome proliferator-activated receptor-gamma coactivator 1-alpha, a key regulator of oxidative phosphorylation (Huiyun and Walter, 2006), to determine whether this metabolic activation and shift towards PDH dependency correlates with changes in oxidative phosphorylation. There was no difference in protein levels of peroxisome proliferator-activated receptor-gamma coactivator 1-alpha between WT and NPC1 cerebellum or associated with AL treatments (Fig. 6D and I).

### ADLL decreases pyruvate dehydrogenase kinase 2 protein expression

PDH is phosphorylated and inactivated by pyruvate dehydrogenase kinase (PDK), reducing flux through the TCA cycle. In contrast, pyruvate dehydrogenase phosphatase (PDP) dephosphorylates and activates PDH, increasing flux through the TCA cycle (Jha *et al.*, 2012). We therefore analysed the expression of three PDK isoforms (PDK1, PDK2 and PDK4) and a single isoform of PDP (PDP1) in the cerebellum (Jha *et al.*, 2012). While levels of PDK1 and PDK4 in the cerebellum were not significantly different between 59-day old *Npc1^−/−^* and *Npc1^+/+^*mice, PDK2 was significantly lower (64.4% reduction compared to wild-type, *P <* 0.0001) (Fig. 6J and K). Furthermore, ADLL (which decreased the phosphorylation of PDH to the greatest extent) decreased PDK2 to a significantly greater extent relative to *Npc1^−/−^* UT group (42.3%, *P =* 0.0022) (Fig. 6J and K). PDP1 was unaltered in untreated *Npc1^−/−^* cerebellum and was only significantly increased by ADL (27.4%, *P =* 0.0433) (Fig. 6J and K).

### ALL decreases superoxide dismutase 1 while ADL increases superoxide dismutase 2

We assessed the impact of ALs on the anti-oxidative system by measuring the cytoplasmic superoxide dismutase 1 (SOD1) and the mitochondrial superoxide detoxifier SOD2. We were unable to detect any significant difference in SOD1 protein levels between *Npc1^−/−^* and *Npc1^+/+^* cerebella but observed a significant reduction in SOD2 (25% *P* = 0.0061) (Fig. 6L and M). We found that only ALL significantly decreased SOD1 levels in cerebellum (15.6%, *P =* 0.0472) and only ADL significantly elevated SOD2 levels when compared to untreated *Npc1^−/−^* mice (19.8%, *P* = 0.0214) (Fig. 6L and M).

### Neuroprotective effects of ADLL in individual-cases of off-label-use in NPC1 patients

**Figure 7:**
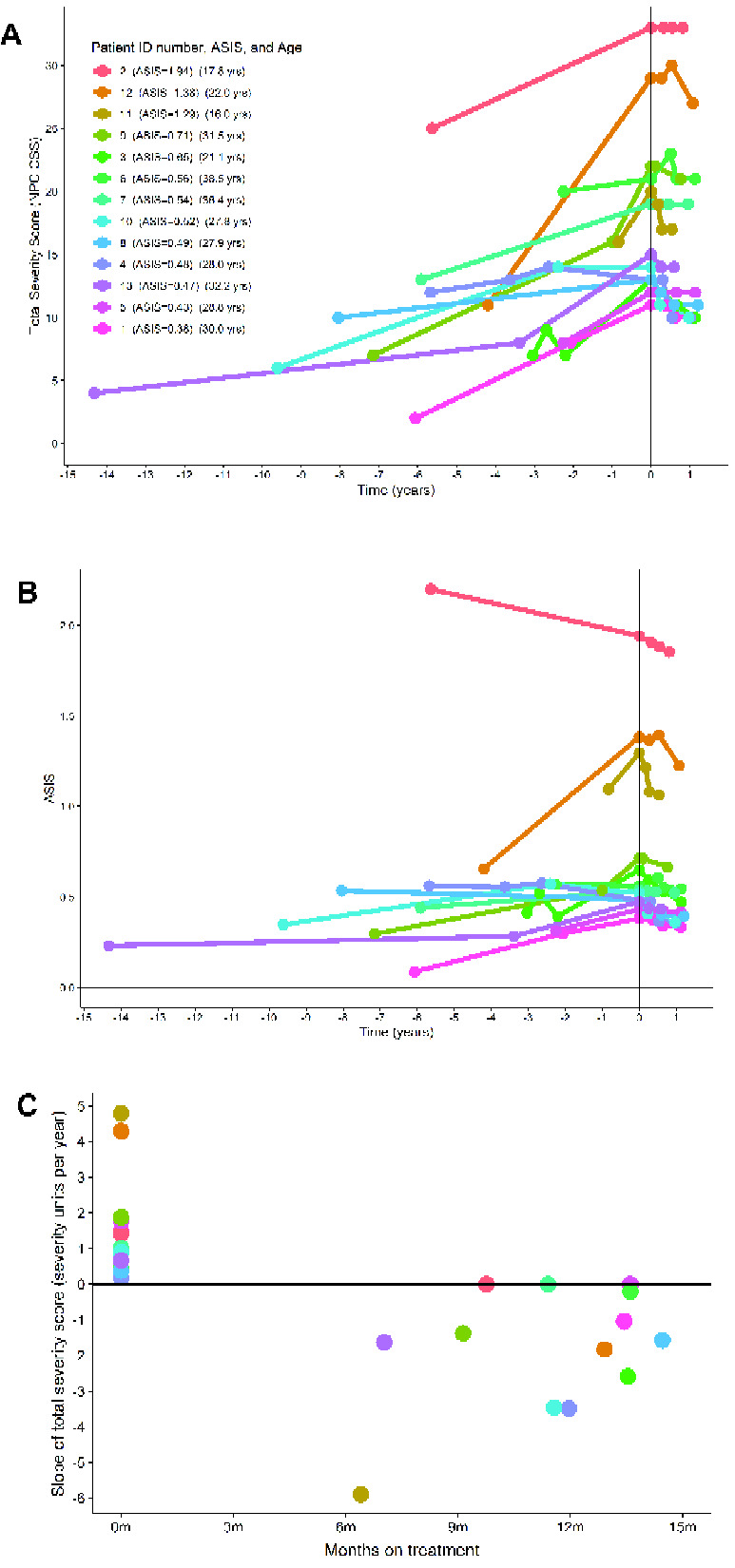
**Clinical data from 13 adult NPC1 patients.** For demographics, see Materials and methods. (**A**) Total clinical severity scores [maximum score 50, with higher values equating to increasing levels of disability (Yanjanin *et al.*, 2010)]. Data were plotted prior to initiation of treatment with Tanganil™ (ADLL), incorporating available retrospective data. (**B**) Clinical data computed as annual severity increment scores (Cortina-Borja *et al.*, 2018) (*P* < 0.001 in annual severity increment score). (**C**) Slope of total severity score (severity units per year) before and after ADLL treatment. Each individual participant in this observational study is colour coded.

Having observed unanticipated beneficial effects of ALs in *Npc1^−/−^* mice we investigated whether neuroprotective effects also occurred in NPC1 patients treated with ADLL enrolled in an observational clinical study (Cortina-Borja *et al.*, 2018) (for demographics of patients see Supplementary Table 2). Total clinical severity scores [with higher values equating to increasing levels of disability (Yanjanin *et al.*, 2010)] were plotted prior to initiation of treatment with Tanganil™ (ADLL), incorporating available retrospective data (Fig. 7A). All 13 patients had positive slopes of disease progression before ADLL treatment (1.8 severity units/year was the average slope). Following initiation of ADLL treatment, the average slope was −1.78 (three patients had no slope, 10 had negative slopes) equating to a significant reduction in disease progression and improvement in the majority of patients (one-sided sign test *P* = 0.0002) (Fig. 7A). The data were also computed as annual severity increment scores (Cortina-Borja *et al.*, 2018) that measure the rate of disease progression. A mean of −9.1%/year (*P* < 0.001) in annual severity increment scores was observed (Fig. 7B). When the slope each patient’s pre-treatment total severity score per year was plotted, all patients had positive slopes, whereas post-treatment slopes were either zero or negative, consistent with stabilization or improvement in disease progression (Fig. 7C). When the treated patient data were analysed by neurological subdomains (Table 1) the majority of patients improved or stabilized on treatment in functional and cognitive subdomains (Table 1).

**Table 1:** Number of patients (total cohort *n* = 13) that improved, stabilized or deteriorated following ADLL treatment stratified by the neurological domains scored (NIH NCSS)

Neurological domains	Improved	Stable	Deteriorated
Eye movement	8	4	1
Ambulation	8	4	1
Speech	3	7	3
Swallow	10	2	1
Fine motor skills	8	3	2
Cognition	6	6	1
Memory	10	2	1

### ADLL shows benefit in other LSDs: effects in Sandhoff mice and GM2 gangliosidosis patients

**Figure 8:**
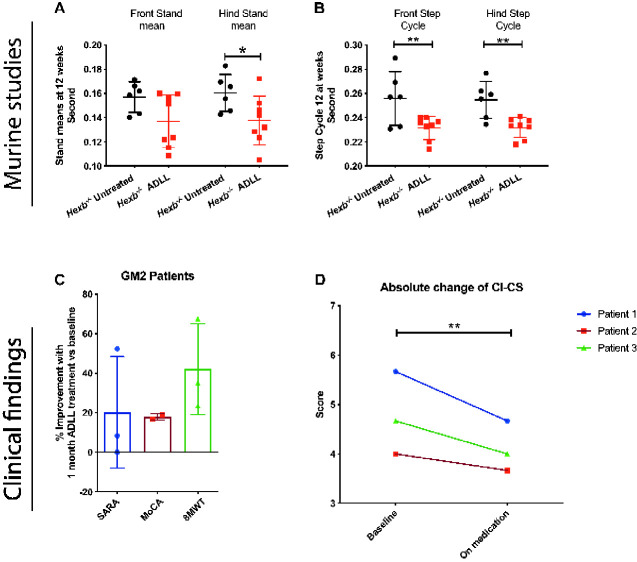
**Effects of ADLL on behavioural and biochemical parameters in 12-week-old Sandhoff disease mice and GM2 patients. Sandhoff untreated (*Hexb^−/−^* UT), ADLL (*Hexb^−/−^* ADLL).** Six to eight animals per group. (**A**) Front-hind stand mean measurements of *Hexb^−/−^* UT and *Hexb^−/−^* ADLL, mean ± SD, *P = 0.0323, two-way ANOVA. (**B**) Front-hind step cycle measurements of *Hexb^−/−^* UT and *Hexb^−/−^* ADLL, mean ± SD, **P < 0.0063, two-way ANOVA. (**C**) Percent improvement in clinical scores in three patients with GM2 gangliosidosis on baseline and after 1 month on medication with ADLL. Scale for Assessment and Rating of Ataxia Montreal Cognitive Assessment and 8-meter-walking-test were assessed. Third patient was excluded from Montreal Cognitive Assessment because the test is not approved for children. See also Videos 1 and 2. (**D**) Unbiased Clinical Impression of Change in Severity scores of GM2 patient videos. Clinical Impression of Change in Severity Skala; 1 = normal, not at all ill; 2 = borderline ill; 3 = mildly ill; 4 = moderately ill; 5 = markedly ill; 6 = severely ill; 7 = among the most extremely ill patients. Paired *t*-test, *P* = 0.0039. For individual scoring data see Table 2.

The Sandhoff (*Hexb^−/−^*) mouse model is pre-symptomatic up to 6–8 weeks of age. Subsequently, they develop tremor and their motor function begins to decline (Jeyakumar *et al.*, 1999). By the later stages of the disease (12–15 weeks), they are inactive and are unable to complete motor function tests such as bar crossing (Jeyakumar *et al.*, 1999). *Hexb^−/−^* animals were treated from weaning with ADLL (0.1 mg/kg/day, the same dose used for treatment of *Npc1^−/−^* mice). ADLL-treated mice had improved gait parameters; including hind stand mean (*P =* 0.0323) (Fig. 8A), front (*P =* 0.0039) and hind step cycle (*P =* 0.0062) (Fig. 8B). To date, ALL and ADL have not been evaluated in the Sandhoff mouse model.

Our findings in Sandhoff mice were extended to individual-cases of off-label-use in three patients with a confirmed GM2 gangliosidosis diagnosis (two Tay-Sachs and one Sandhoff disease) [the latter case in press (Bremova-Ertl *et al.*, 2020)] treated with ADLL. We found a mean improvement of the Scale for Assessment and Rating of Ataxia by 20.3%, the Montreal Cognitive Assessment by 17.8% and the 8-meter-walking-test by 42%, as shown in Fig. 8C. All patients and caregivers also reported a subjective improvement and have continued treatment at the same dosage. Videos of the effect of treatment on gait and postural instability are shown in Videos 1 and 2. In addition, three experienced movement disorder experts performed blinded analysis of the videos and rated the videos based on the Clinical Impression of Change in Severity (1 = normal, not at all ill; 2 = borderline ill; 3 = mildly ill; 4 = moderately ill; 5 = markedly ill; 6 = severely ill; 7 = among the most extremely ill). The unbiased observation before and after ADLL treatment showed statistically significant improvements on overall scoring (*t*-test *P* = 0.0039) (Fig. 8D and Table 2). ALL and ADL have not been evaluated in clinical settings, but trials with ALL are being conducted [NPC (NCT03759639) and GM2 gangliosidosis (NCT03759665) and Ataxia-Telangiectasia (NCT03759678)].

**Table 2:** Blinded scoring of GM2 gangliosidosis patient videos pre- and post-ADLL treatment

Examiner number	Patient 1 baseline	Patient 1 treated	Patient 2 baseline	Patient 2 treated	Patient 3 baseline	Patient 3 treated	Paired *t*-test
1	5	4	3	3	3	3	
2	6	5	4	3	5	4	
3	6	5	5	5	6	5	
Mean	5.67	4.67	4	3.67	4.67	4	*P* = 0.0039

Clinical Impression of Change in Severity Skala: 1 = normal, not at all ill; 2 = borderline ill; 3 = mildly ill; 4 = moderately ill; 5 = markedly ill; 6 = severely ill; 7 = among the most extremely ill patients.

## Discussion

In this study, we have investigated the effects of AL in mouse models of NPC1 and Sandhoff disease and in patients to better understand its therapeutic potential and to gain insights into its MOA.

We found that ADLL, ALL and ADL significantly improved ataxia when symptomatic *Npc1^−/−^* mice were treated acutely for 7 days; this is in agreement with observational studies in NPC1 patients (Bremova *et al.*, 2015) treated with ADLL, using the same dosage per kg and day. The individual enantiomers provided similar benefit to the racemic mixture for the symptomatic treatment of ataxia. The MOA explaining how ADLL and the enantiomers improve symptoms remains unknown. All AL analogues tested reduced lipid storage in neuronal and non-neuronal tissues in *Npc1^−/−^* mice and in CHO cells null for *NPC1* suggesting a MOA that is not confined to neuronal cells. The AL analogues were also observed to differentially reduce the levels of stored lipids in the liver and, to a lesser extent, in the brain of treated *Npc1^−/−^* mice. The mechanism that underpins this ‘substrate reduction’ action of ALs currently remains unclear, but in view of the high degree of synergy when combined with the substrate reduction therapy drug miglustat, it may not be a major contributor to AL’s therapeutic effect in NPC1 disease.

Another major finding of the current study was that ADLL and ALL (but not ADL) slowed disease progression when treatment was initiated before symptom onset, consistent with a neuroprotective mechanism. Since the neuroprotective effects were only observed with ADLL and ALL, this implicated ALL as the active enantiomer and demonstrates that the symptomatic improvement in ataxia and neuroprotection are achieved through different MOA. ALL significantly reduced neuroinflammation, which is important as this actively contributes to disease progression and reducing inflammation using nonsteroidal anti-inflammatory drugs has previously been shown to be beneficial in *Npc1^−/−^* mice (Williams *et al.*, 2014).

The neuroprotective effect of ADLL and ALL prompted us to determine whether similar effects occur in NPC1 patients treated with ADLL. We took advantage of an ongoing observational study in which 13 NPC1 patients have been treated with Tanganil™ (ADLL) continuously for ∼1 year and found that all patients showed stabilization or improvement in clinical scores, which were across all neurological domains, not just those relating to ataxia, supporting a more global neuroprotective effect in patients, analogous to those observed in the *Npc1^−/−^* mouse.

One central question arising from these studies is the nature of the underlying MOA and in the case of ADLL and ALL, neuroprotective benefit in NPC1. Therefore, we investigated aspects of cell biology and metabolism known to be sensitive to leucine. Leucine has been shown to activate mTOR and reduce LC3-II and p62 in NPC1 cells (Yanagisawa *et al.*, 2017). However, in this study, ADLL, ALL and ADL did not significantly affect either mTOR or autophagy in *Npc1^−/−^* mouse cerebellum. This might be due to the presence of the acetyl group, as opposed to a primary amine in leucine, blocking the interaction with mTOR, shown in HeLa cells (Nagamori *et al.*, 2016) or that AL analogues distribute at the cellular level in a manner that prevents their interaction with mTOR. Catabolism of leucine serves as a source of acetyl-CoA (Harris *et al.*, 1985), which activates mTOR and nutrient sensing pathways (Son *et al.*, 2019). However, the NPC1 cerebellum has significantly decreased levels of acetyl-CoA (Kennedy *et al.*, 2016). This low acetyl-CoA content in NPC1 might therefore prevent nutrient sensing pathway activation. The complexity of mTOR pathways and altered metabolism more generally in NPC1 makes it likely that the effects of AL will likely be context specific.

Altered glucose/energy metabolism has previously been documented in the pre-symptomatic *Npc1^−/−^* mouse cerebellum (Kennedy *et al.*, 2013). PDHE1 alpha was found to be inactivated and there was a shift from pyruvate/PDH dependency (upon which neurons rely for aerobic respiration) towards lactate/LDH (i.e. anaerobic respiration). The consequence of this would be progressive impairment of energy generation (Kennedy *et al.*, 2013). In this same study, alterations in cerebellar amino acids (including leucine) and low acetyl-CoA were also reported (Kennedy *et al.*, 2013). Independent metabolomics studies on *Npc1^−/−^* mouse liver reported imbalances in amino acid levels (Ruiz-Rodado *et al.*, 2016).

In view of data implicating altered energy metabolism in *Npc1^−/−^* mice, we measured ADP/ATP and NAD/NADH ratios, as a sensitive measure of energy status (Stein and Imai, 2012; Murphy and Hartley, 2018) and found that ADLL treatment might have shifted the system towards a more TCA-dependent glycolytic state, in the absence of significant changes to NAD-NADH coenzyme levels (which are lower in *Npc1^−/−^* cerebellum), in agreement with previous findings in *Npc1^−/−^* liver (Ruiz-Rodado *et al.*, 2016). We then studied whether the shift in glycolysis is towards pyruvate utilization to produce lactate (anaerobic pathway) or to produce acetyl-CoA (aerobic pathway). We found that ADLL treatment significantly increased PDHE1 alpha levels, and decreased PDH enzyme phosphorylation (causes enzyme inactivation) in the cerebellum suggesting activation of the aerobic pathway. Interestingly, PDH-deficient mice also display Purkinje neuron degeneration and relapsing ataxia (Pliss *et al.*, 2013), suggesting the ability of ADLL to protect Purkinje cells against degeneration may be via activation of PDH. However, our findings are based on whole brain lysate and more detailed studies on discreet cell populations would be informative. This effect on PDH did not reflect a change in PGC1-alpha protein levels (indicator of oxidative phosphorylation, OXPHOS), which might be due to the fact that up-regulating OXPHOS requires coenzyme NADH and ADLL treatment does not increase NADH levels.

**Figure 9:**
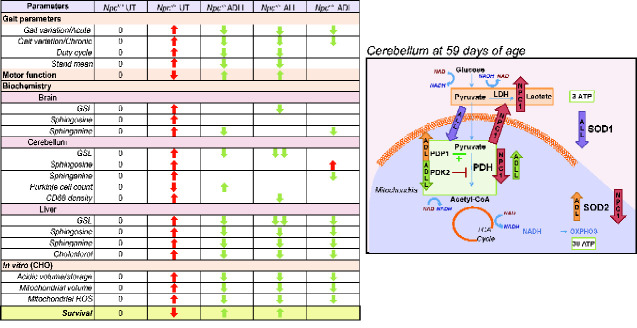
**Summary table.** Summary table of the effect of AL treatments on investigated pathologies *in vivo*, *ex vivo* and *in vitro*, along with the proposed mechanism of AL treatments in the cerebellum. Red arrows indicate the pathologies in NPC1 relative to its wild-type counterparts. Green arrows indicate the significant changes in response to AL treatment.

Individual enantiomers had distinct effects in NPC1 cerebellum; while ALL normalizes altered levels of PDH and LDH and mildly reduces SOD1 levels, ADL enhanced levels of the mitochondrial reactive oxygen species scavenger SOD2 (manganese dependent SOD2). The biological relevance of subtle change in SOD1 level with ALL is unknown and functional readouts are needed to validate this result. *In vivo*, ADLL and ALL treatments were associated with a significantly greater benefit in motor function and lifespan relative to ADL (Fig. 1). This study suggests that targeting altered cellular metabolism in neurodegenerative diseases may achieve greater efficacy than using antioxidant approaches. The metabolic changes in *Npc1^−/−^* mice in response to AL treatments are summarized in Fig. 9.

Finally, we explored whether the benefits of AL extend to other LSDs. We observed efficacy of ADLL in a mouse model of Sandhoff disease, one of the GM2 gangliosidoses (Platt *et al.*, 2012), a subgroup of GSL lysosomal storage diseases (LSDs) that includes Tay-Sachs disease: we demonstrated that ADLL-treated Sandhoff mice had a significant increase in lifespan and improvements in motor function (Kaya *et al.*, 2020); however, the individual isomers ALL and ADL were not assessed. We also found improvement in gait parameters in *Hexb^−/−^* mice upon ADLL therapy consistent with the observational clinical studies we conducted. In three patients with GM2 gangliosidosis with ataxia that were treated with ADLL in individual-cases of off-label-use gait significantly improved, supporting the concept that ADLL and ALL may also be efficacious in multiple LSDs and other neurodegenerative diseases.

In conclusion, we have found that a well-tolerated drug, ADLL, and its enantiomer ALL slow disease progression and improve motor function and lipid accumulation in murine and cell models of NPC1. Furthermore, in observational studies in NPC1 patients, treatment with ADLL was associated with improvement in multiple neurological domains and a significant reduction in the rate of disease progression, potentially via restoration of aerobic metabolism based on the mechanistic studies conducted in the *Npc1^−/−^* mouse model. The striking synergy of ADLL with miglustat in *Npc1^−/−^* mice suggests that combining these two drugs will lead to improved short- and long-term clinical outcomes and merits clinical trials. It is interesting to note that 12 of the 13 NPC1 patients in the observational clinical study were on the standard of care miglustat, so part of their clinical improvement may be the result of the synergy between the two disease-modifying drugs. The individual enantiomers ALL and ADL have not yet been tested in combination with miglustat. The finding that both a mouse model of Sandhoff disease and GM2 gangliosides patients, regardless of age of onset, also showed clinical improvement when treated with ADLL suggest that this, and related analogues, may have broader utility in LSDs and more common neurodegenerative diseases. Based on ALL being the neuroprotective enantiomer, clinical trials with ALL are currently being conducted [NPC (NCT03759639), GM2 gangliosidosis (NCT03759665) and Ataxia-Telangiectasia (NCT03759678)].

## Supplementary material

Supplementary material is available at *Brain Communications* online.

## Supplementary Material

fcaa148_Supplementary_Data

## Abbreviations

ADL =: acetyl-d-leucine
ADLL =: acetyl-dl-leucine
ADP =: adenosine diphosphate
AL =: acetyl-leucine
ALL =: acetyl-l-leucine
BCKDHA =: branched chain keto acid dehydrogenase subunit A
CHO =: Chinese hamster ovary
GSL =: glycosphingolipid
LDH =: lactate dehydrogenase
LSD =: lysosomal storage disease
MOA =: mechanism of action
mTOR =: mammalian target of rapamycin
NAD =: nicotinamide adenine dinucleotide
NADH =: nicotinamide adenine dinucleotide (reduced form)
NPC1 =: Niemann-Pick disease type C1
PDH =: pyruvate dehydrogenase
PDK =: pyruvate dehydrogenase kinase
PDP =: pyruvate dehydrogenase phosphatase
SOD =: superoxide dismutase
TCA =: tricarboxylic acid
