# Supplementary material for: Acetyl-leucine slows disease progression in lysosomal storage disorders
Source: Brain Commun. 2020 Dec 20;3(1):fcaa148. doi: 10.1093/braincomms/fcaa148 (PMC7954382; doi:10.1093/braincomms/fcaa148)
Supplement: fcaa148_Supplementary_Data [file fcaa148_Supplementary_Data.zip › Supplementary_Table_2.docx]

| **Patient ID** | **Age** | **Country of origin** | **Age onset** | **Age diagnosis** | **Sex** | **Mutation** | **Miglustat (yes/no) treatment, [months]** | **Acetyl-DL-Leucine treatment, [months]** |
| --- | --- | --- | --- | --- | --- | --- | --- | --- |
| 1 | 32 | Germany | 4 | 24 | M | *NPC1*: c.709C>T(p.Pro237Ser (de novo). 2. mutation not found | No | 12 |
| 2 | 20 | Germany | 0.75 | 1 | M | *NPC1:* c.2861C>T (p.S954L). c.2861C>T (p.S954L) Homozygot | 102 | 10 |
| 3 | 23 | Germany | 10 | 11 | F | Homozygot c.3246-5_3246-7del (novel mutation described in Bremova-Ertl T. 2020)^1^ | 90 | 12 |
| 4 | 30 | Germany | 10 | 21 | M | *NPC1:* c.2474A.G. c.3160G.A | 100 | 12 |
| 5 | 31 | Germany | 21 | 23 | M | *NPC1:* c.2660C>T c.3019C>G | 70 | 12 |
| 6 | 41 | Germany | 12 | 33 | M | *NPC1:* c.2660C>T c.3019C>G | 70 | 12 |
| 7 | 39 | Germany | 16 | 15 | F | *NPC1:* c.2776G>A (p.Ala926Thr). c.2861C>t (p.Ser954Leu) | 117 | 12 |
| 8 | 30 | Germany | 16 | 19 | M | *NPC1:* c.2195insT. c.2474A>G (Y825C) | 127 | 12 |
| 9 | 34 | Germany | 18 | 20 | M | *NPC1:* c. 2861C>T [S954L]. 3433t>C [W1145R] | 78 | 12 |
| 10 | 30 | Slovakia | 14 | 15 | M | *NPC1:* c.1723delG. c.2861C.T | 118 | 21 |
| 11 | 18 | Saudi Arabia | 8 | 8 | M | *NPC1:* c.2974G>t. p.G992W | 61 | 6 |
| 12 | 24 | Sweden | 9 | 10 | F | *NPC1*: c. [1211G>A];[1843C>T]. p.[Arg404Gln];[Arg615Cys] | 90 | 13 |
| 13 | 34 | Germany | 1 | 17 | M | *NPC1*: Homozygot c.387T>C. c.3182T>C (Ile1061Thr). | 78 | 12 |

1. Bremova-Ertl, T. *et al.* Clinical, ocular motor, and imaging profile of Niemann-Pick type C heterozygosity. *Neurology* (2020) doi:10.1212/WNL.0000000000009290.

**Supplementary Table 2.** Demographics of 13 patients under longitudinal treatment with acetyl-DL-leucine.
