# Supplementary material for: Acetyl-leucine slows disease progression in lysosomal storage disorders
Source: Brain Commun. 2020 Dec 20;3(1):fcaa148. doi: 10.1093/braincomms/fcaa148 (PMC7954382; doi:10.1093/braincomms/fcaa148)
Supplement: fcaa148_Supplementary_Data [file fcaa148_Supplementary_Data.zip › FL_Western_blots.pdf]

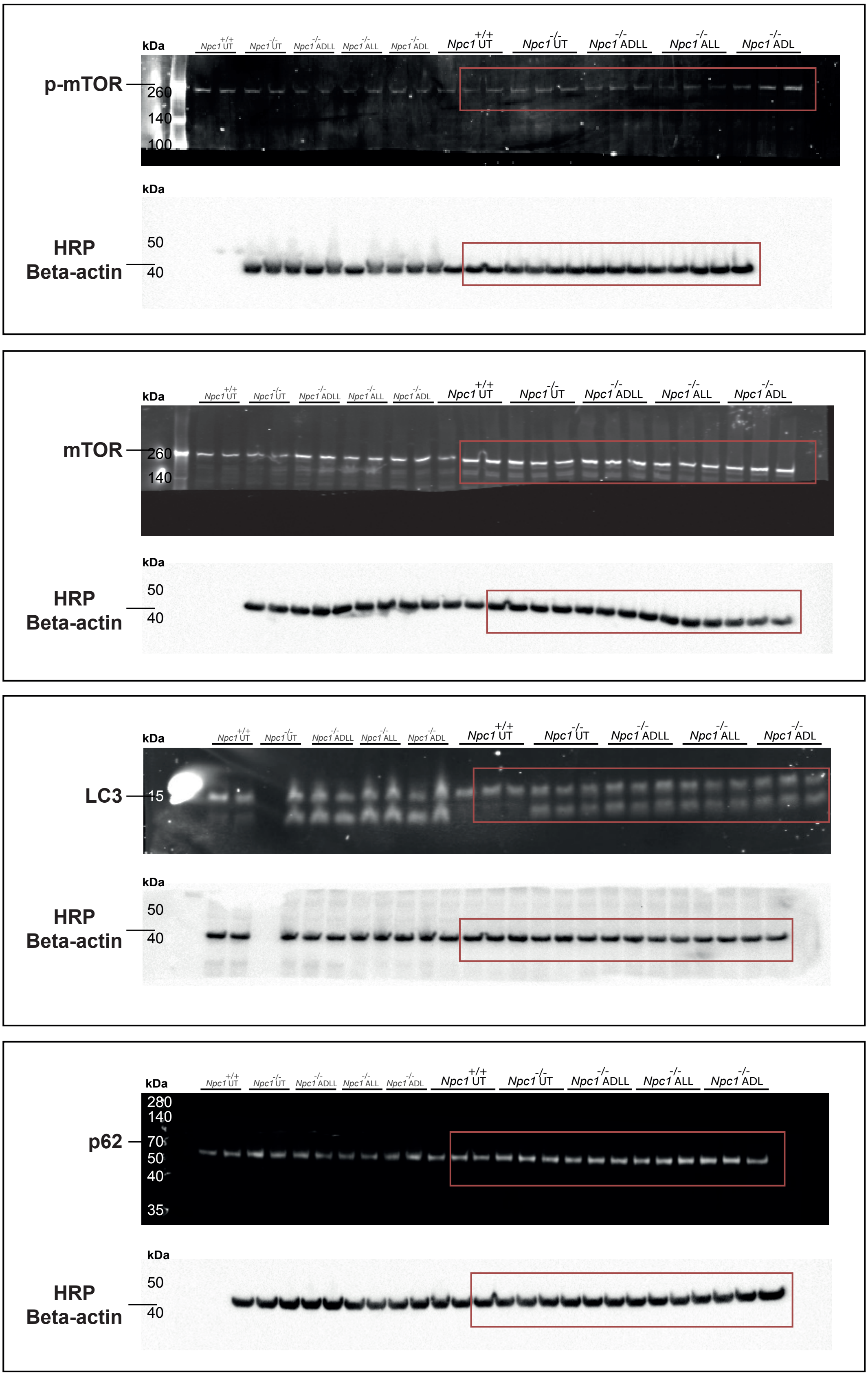

Figure 5 D full-length western blots

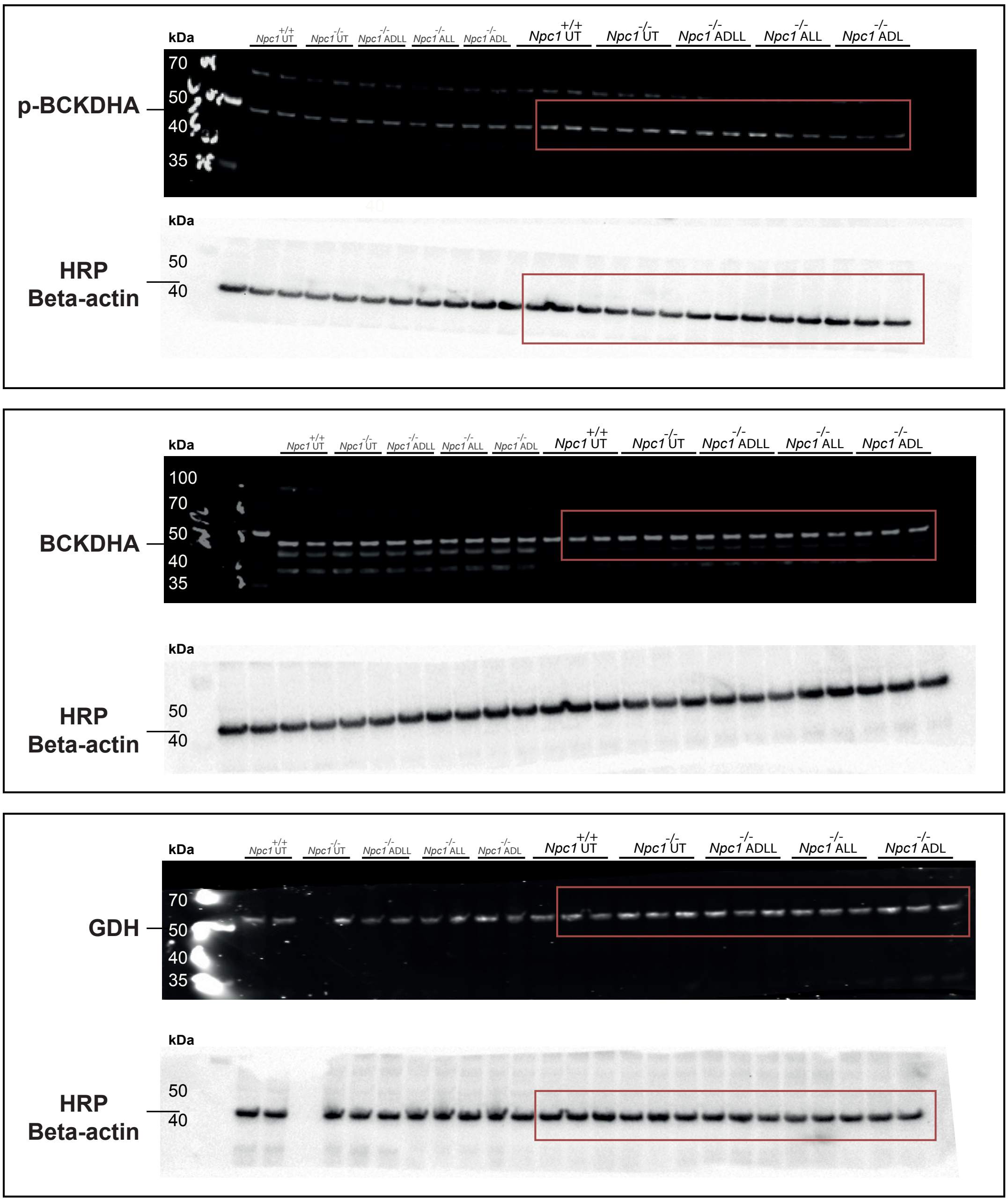

Figure 5 F full-length western blots

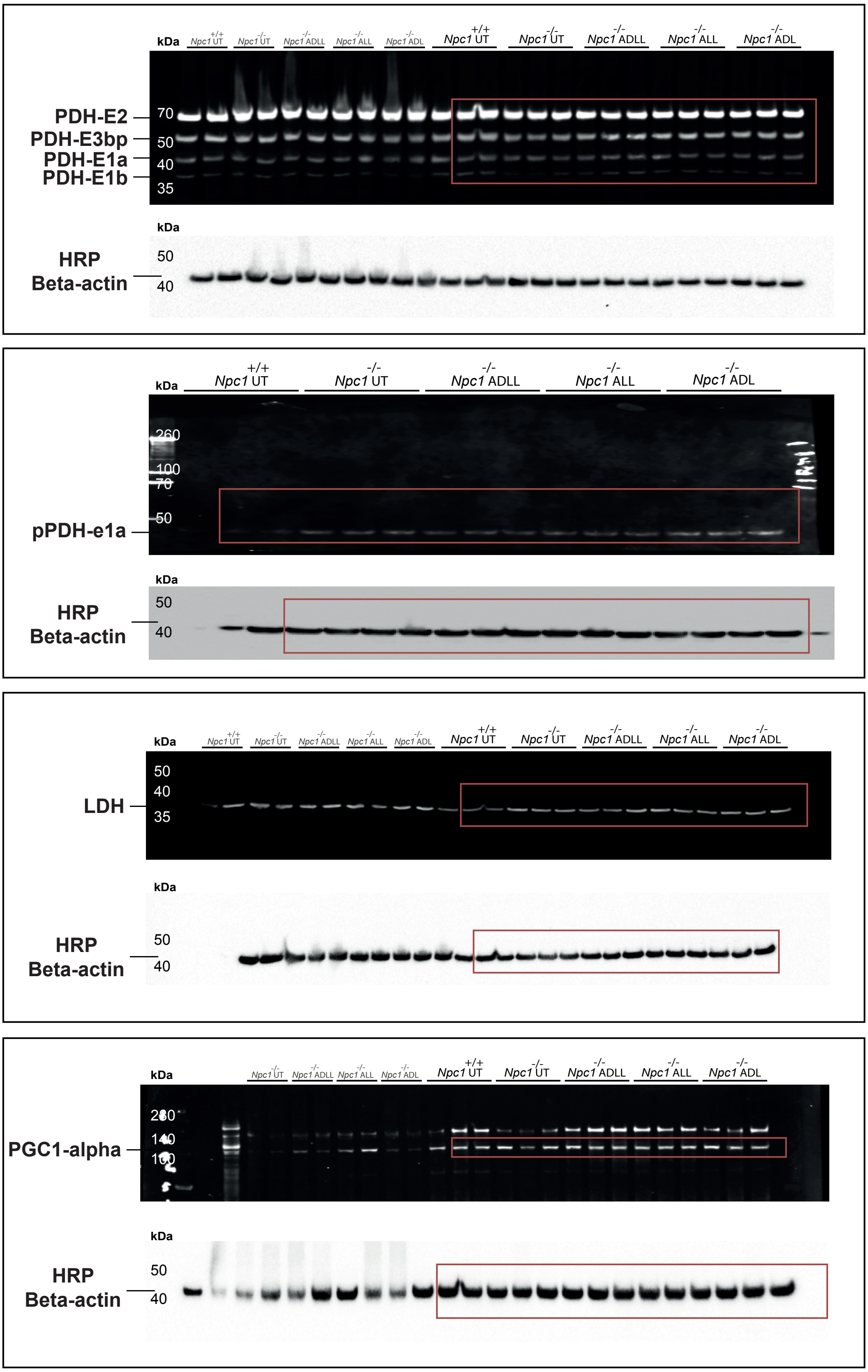

Figure 6 D full-length western blots

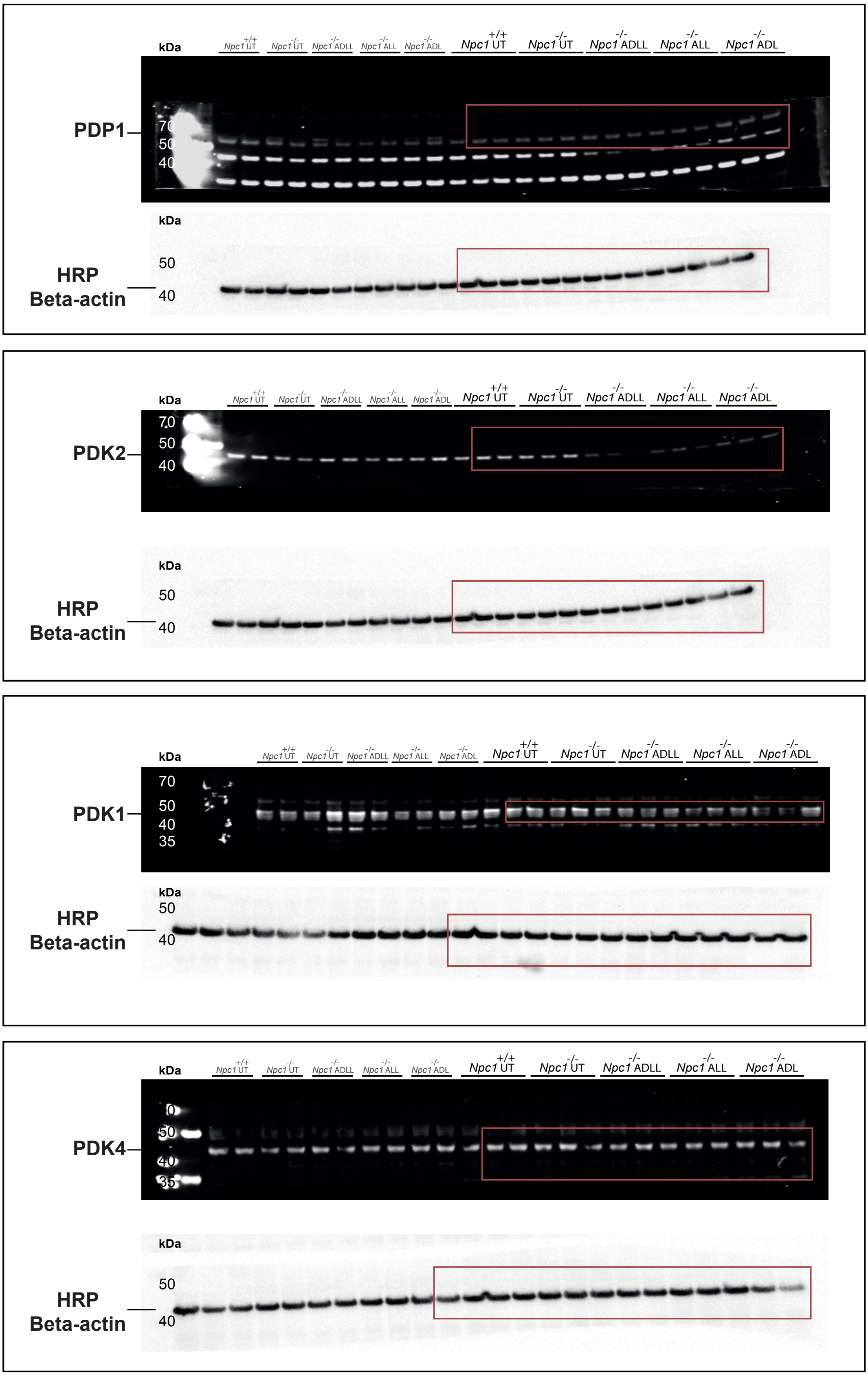

Figure 6 K full-length western blots

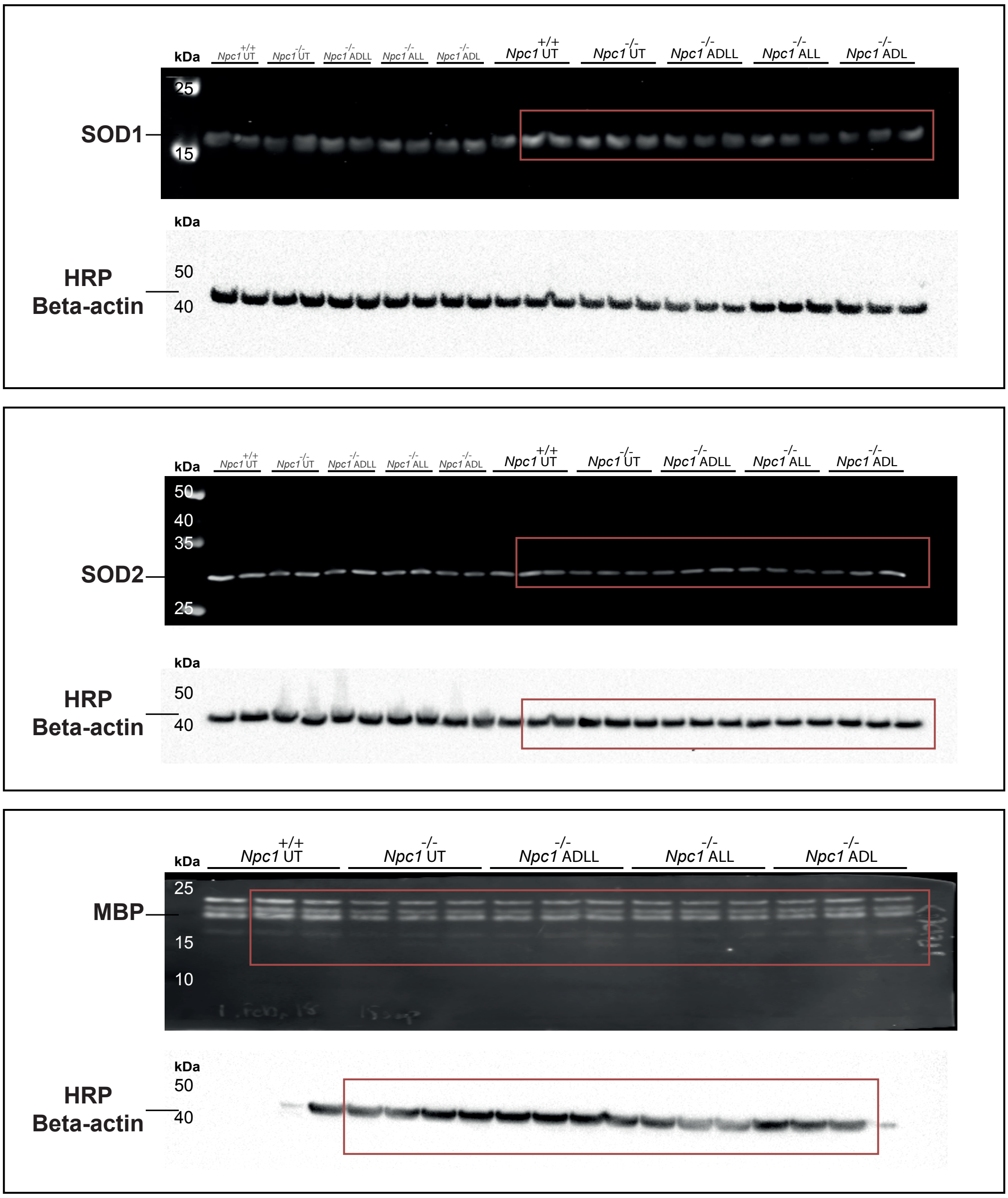

Figure 6 M and Supplementary Figure 2 F full-length western blots
