# Supplementary material for: Acetyl-leucine slows disease progression in lysosomal storage disorders
Source: Brain Commun. 2020 Dec 20;3(1):fcaa148. doi: 10.1093/braincomms/fcaa148 (PMC7954382; doi:10.1093/braincomms/fcaa148)
Supplement: fcaa148_Supplementary_Data [file fcaa148_Supplementary_Data.zip › Supplementary_Figures.pdf]

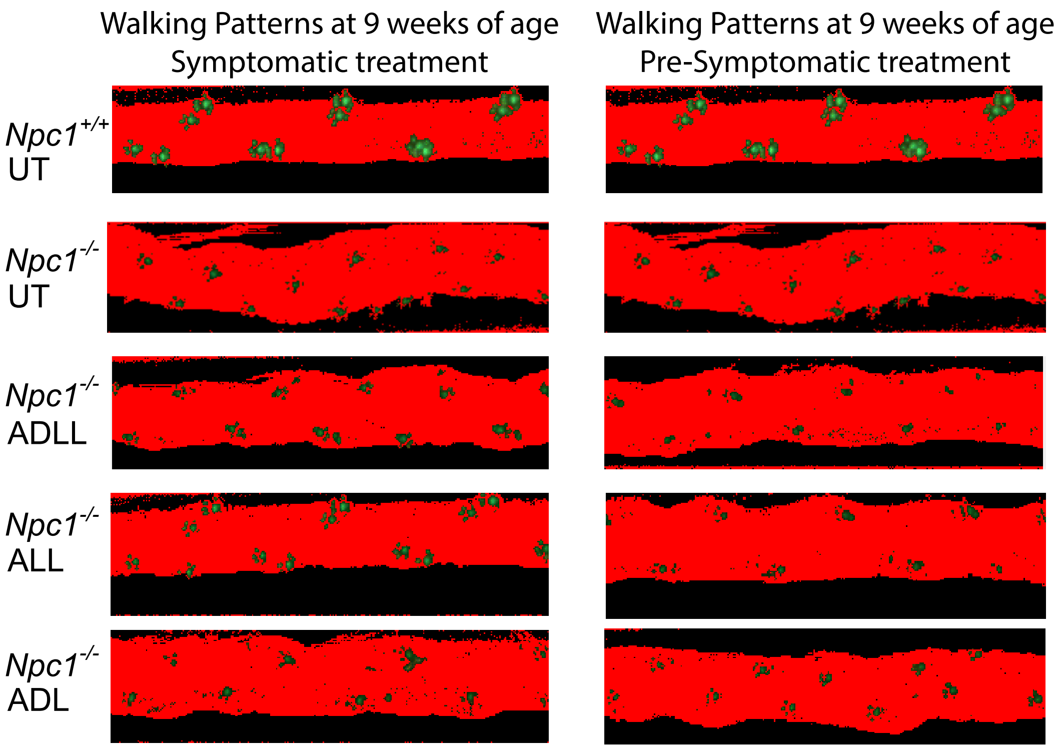

Supplementary Figure 1. Gait of mice with AL treatments initiated in the symptomatic or pre-symptomatic period. Heat maps of mice which display the body route and footprints was obtained by Noldus Catwalk software. Red indicates the heat of the mouse body.

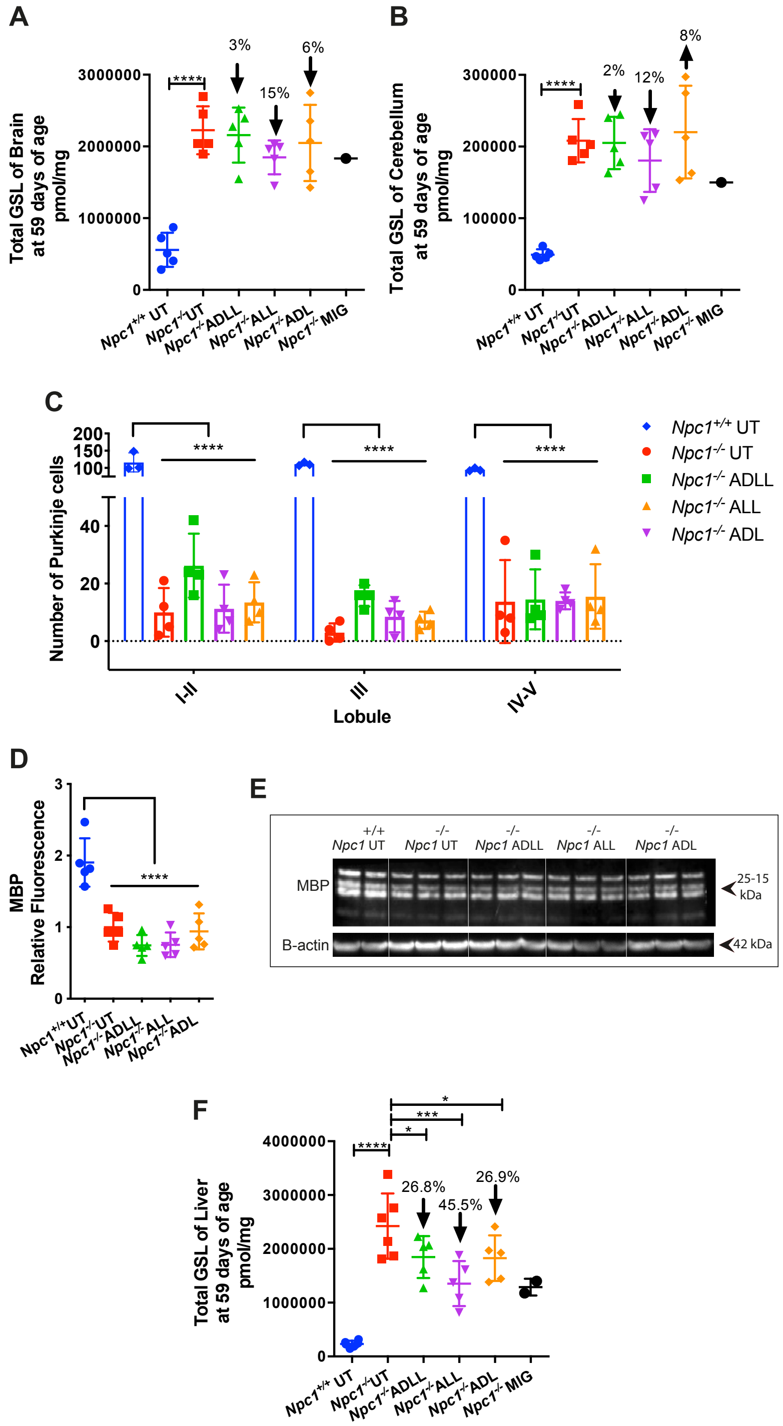

Supplementary Figure 2. Effect of Acetyl leucine analogues on biochemical and histopathology in *Npc1*$^{-/-}$ mice. For wild type untreated (*Npc1*$^{+/+}$ UT), NPC1 untreated (*Npc1*$^{-/-}$ UT), ADLL (*Npc1*$^{-/-}$ ADLL), ALL (*Npc1*$^{-/-}$ ALL), ADL (*Npc1*$^{-/-}$ ADL) treatments n= 5 animals per group, miglustat treatment (*Npc1*$^{-/-}$ MIG) n= 2 or n= 1. **A)** Total GSL measurements in forebrain (brain), Mean ± SD, ****$p< 0.0001$ (One-way ANOVA). **B)** Total GSL measurements in cerebellum, Mean ± SD, ****$p< 0.0001$ (One-way ANOVA). **C)** Purkinje cell counts in the mouse cerebellum

lobules 1 to 5. N=3 for *Npc1*$^{+/+}$ UT, and n=4 for *Npc1*$^{-/-}$ UT, ADLL, ALL, ADL groups. Mean ± SD, ****p<0.0001 (2-way ANOVA). **D)** Fluorescence quantification of cerebellar MBP at 59 days of age, relative to NPC untreated group normalised to beta-actin. Mean ± SD, **** p<0.0001 (One-way ANOVA). **E)** Western blot illustrative image of MBP expression and beta-actin loading controls. **F)** Total GSL measurements in liver, Mean ± SD, *p<0.034, ***p= 0.0004, ****p<0.0001 (One-way ANOVA).

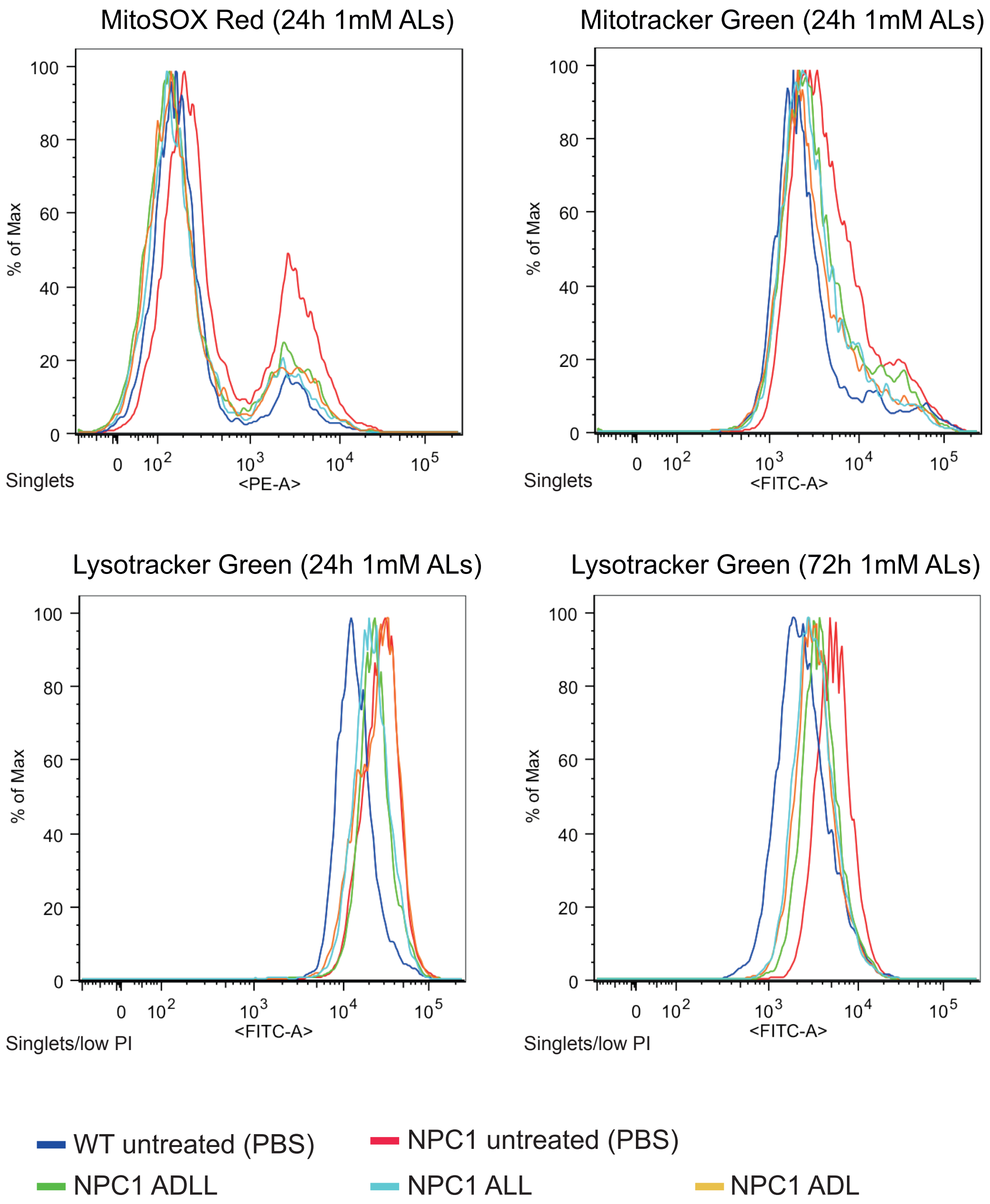

Supplementary Figure 3. Flow cytometry traces of CHO cells and AL treatments. Cells were either treated with 1mM ALs (in PBS) or the same volume of PBS and stained for mitochondrial ROS (MitoSOX Red) or membrane potential-independent mitochondrial volume (MitoTracker Green). Total acidic compartment of the cells was determined with LysoTracker Green, co-stained with propium iodide (PI).
