# Supplementary material for: Acetyl-leucine slows disease progression in lysosomal storage disorders
Source: Brain Commun. 2020 Dec 20;3(1):fcaa148. doi: 10.1093/braincomms/fcaa148 (PMC7954382; doi:10.1093/braincomms/fcaa148)
Supplement: fcaa148_Supplementary_Data [file fcaa148_Supplementary_Data.zip › Supplementary_Table_1_Antibody_list.docx]

| *Abv/acronym* | *Antibody type* | *Host* | *Source* | *Catalogue code* | *Dilution* | *ug protein loaded* |
| --- | --- | --- | --- | --- | --- | --- |
| Calbindin-D28K | Mab | Rabbit | Swant | CB38a | 1:2000 | NA |
| CD68 | Mab | Rat | Bio-Rad | MCA1957 | 1:500 | NA |
| MBP | Mab | Mouse | Biolegend | 836504 | 1:2000 | 10 ug |
| Alexa Fluor 594 anti rabbit | Secondary Ab | Goat | abcam | ab150080 | 1:1000 | NA |
| Alexa Fluor 488 anti rat | Secondary Ab | Goat | abcam | ab150157 | 1:2000 | NA |
| LC3B | Polyclonal | Rabit | abcam | ab51520 | 1:2000 | 10 ug |
| P62 | Mab | Mouse | abcam | ab56416 | 1:2000 | 10 ug |
| GDH | Mab | Rabit | CellSignalling | D9F7P | 1:1000 | 15 ug |
| pMTOR (ser 2448) | Polyclonal | Rabit | CellSignalling | 2971 | 1:500 | 30 ug |
| mTOR | Polyclonal | Rabit | CellSignalling | 2972 | 1:500 | 30 ug |
| PDH Complex | Polyclonal | Mouse | abcam | ab110416 | 1:1000 | 25 ug |
| pPDH (s293) | Polyclonal | Rabit | abcam | ab92696 | 1:500 | 25 ug |
| SOD1 | Polyclonal | Rabit | abcam | ab13498 | 1:1500 | 15 ug |
| SOD2 | Polyclonal | Rabit | abcam | ab56416 | 1:1500 | 15 ug |
| PDP1 | Polyclonal | Rabit | abcam | ab228578 | 1:1000 | 25 ug |
| PDK1 | Mab | Mouse | abcam | ab110025 | 1:1000 | 25 ug |
| PDK2 | Mab | Rabit | abcam | ab68164 | 1:1000 | 25 ug |
| PDK4 | Mab | Rabbit | abcam | ab214938 | 1:1000 | 25 ug |
| BCKDH-A | Polyclonal | Rabbit | abcam | ab90691 | 1:1000 | 25 ug |
| pBCKDH-A | Polyclonal | Rabbit | abcam | ab200577 | 1:1000 | 25 ug |
| LDHB | Mab | Mouse | abcam | ab85319 | 1:1500 | 25 ug |
| PGC-1 alpha | Mab | Mouse | Merck Millipore | ST1202 | 1:1500 | 25 ug |
| Anti-Mouse 800CW IgG (H + L) | Secondary Ab | Goat | Licor-IRDye | 925-32210 | 1:10,000 | NA |
| Anti-Rabbit 680RD IgG (H + L) | Secondary Ab | Goat | Licor-IRDye | 925-68071 | 1:10,000 | NA |
| HRP conjugated beta actin | Mab | Mouse | Invitrogen | MA5-15739-HRP | 1:15,000 | NA |
| Pierce ECL Substrate Kit | - | - | Thermo Fisher | 32106 | - | NA |

**Supplementary Table 1.** List of antibodies /reagent used for immunohistochemistry and western blotting.
